# Impedance Spectroscopy as a Powerful Tool for Researching Molybdenum-Based Materials with Schiff Base Hydrazones

**DOI:** 10.3390/ma16031064

**Published:** 2023-01-25

**Authors:** Josipa Sarjanović, Martina Stojić, Mirta Rubčić, Luka Pavić, Jana Pisk

**Affiliations:** 1 Department of Chemistry, Faculty of Science, University of Zagreb, Horvatovac 102a, 10000 Zagreb, Croatia; 2 Ruđer Bošković Institute, Bijenička Cesta 54, 10000 Zagreb, Croatia

**Keywords:** molybdenum, Schiff base, aroylhydrazone, impedance spectroscopy, structural transformations

## Abstract

Molybdenum coordination complexes are widely applied due to their biological and pharmacological potential, as well as their performance in different catalytic processes. Parent dioxidomolybdenum Schiff base complexes were prepared via the reaction of [MoO_2_(acac)_2_] with a hydrazone Schiff-base tetradentate ligand. A new hydrazone-Schiff base (H_2_L^1 and 2^) and its corresponding mononuclear and polynuclear dioxidomolybdenum(VI) complex were synthesized and characterized by spectroscopic methods and elemental analyses, and their thermal behavior was investigated by thermogravimetry. The crystal and molecular structures of H_2_L^2^ ligands and the complexes [MoO_2_(L^1^)(H_2_O)], [MoO_2_(L^2^)(H_2_O)], [MoO_2_(L^1^)(MeOH)]∙MeOH, [MoO_2_(L^1^)(EtOH)]∙EtOH, [MoO_2_(L^1^)(2-PrOH)]∙2-PrOH, and [MoO_2_(L^1^)]*_n_* were determined by single-crystal X-ray diffraction. Using the in situ impedance spectroscopy method (IS), the structural transformations of chosen complexes were followed, and their electrical properties were examined in a wide range of temperatures and frequencies.

## 1. Introduction

**Scheme 1:**
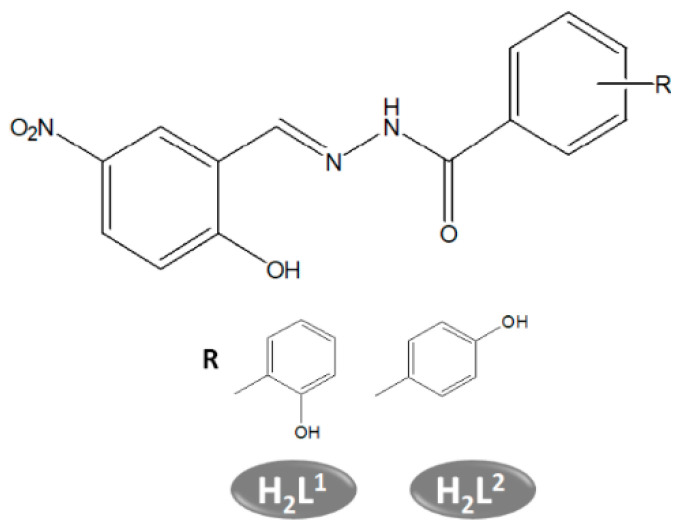
Hydrazone ligands used for the preparation of Mo complexes.

The principal interest in the chemistry of molybdenum(VI) complexes with Schiff-base ligands arises from their biological and pharmacological importance [1,2]. To a greater extent, molybdenum compounds have been widely applied and researched in different catalytic processes, such as sulfoxidation [3,4], olefin epoxidations [5,6,7], alcohol oxidations [8], etc. The capability of molybdenum to adapt and easily convert between different oxidation states signals further applications. For instance, it has been shown that transition metal complexes with Schiff bases have potential in electrochemical or molecular materials with nonlinear optical properties [9]. Furthermore, vanadium complexes with quadridentate Schiff bases demonstrate semiconducting properties [10]. Schiff-base N,N′-bis-(furaldehyde)-1,2-phenylenediamine-dipicolyl metallic complexes of Cu(II), Co(II), and Hg(II), and the N,N′-bis-(furaldehyde)-1,2-ethylenediamine-dipicolyl Cu(II) complex, incorporated into a plasticized PVC membrane, were used to prepare highly selective thiocyanate electrodes. The mechanism for selectivity towards SCN^−^ was supported by AC impedance measurements [11]. Dielectric and conductivity properties of Co(II), Cu(II), Ni(II), and Cd(II) complexes of azines derived from benzophenonehydrozone with different aldehydes were reported [12], as well as the conducting performance of Co(II), Ni(II), and Cu(II) derived from imidazole-2-carboxaldehyde and glycylglycine [13]. Furthermore, Joseyphus et al. reported on the dielectric properties and conductivity studies of tetradentate Co(II), Cu(II), and Ni(II) Schiff-base complexes [14]. Based on the above studies, the research aims of the present study were to prepare a new class of molybdenum materials containing Schiff-base ligands and to study them further by impedance spectroscopy. To the best of our knowledge, the electric and conducting properties of these kinds of molybdenum materials have not been reported so far, making this investigation novel and promising. The Schiff-base ligands used for the preparation of the molybdenum complexes are presented in Scheme 1. The ligands were chosen based on our previous studies with similar types of ligands in order to correlate the substituent on the aldehyde ring and its potential influence on the type of coordination complex [15]. Furthermore, the Cambridge Structural Database (CSD 5.43, with updates up to September 2022) revealed, besides the structure of H_2_L^1^ ligand (CSD, ref ADEWOW) [16], only two examples of coordination compounds with the ligands used in this study: H_2_L^1^ with a Ni metal center (CSD, ref BOTPIK) [17], and H_2_L^2^ with a Zn metal center, (CSD, ref RAPZAM) [18]. In order to enrich the chemistry of molybdenum, the present study is focusses on molybdenum mononuclear and polynuclear complexes with the above-mentioned types of ligands.

## 2. Materials and Methods

### 2.1. Preparation

The starting compounds used commercially available aldehydes and hydrazide (Aldrich, St. Louis, MO, USA), as well as the solvents MeCN and MeOH (Fluka), without any purification. The starting complexes [MoO_2_(acac)_2_] (acac = C_5_H_7_O_2_ = acetylacetonate) were prepared as described in the literature [19,20].

#### 2.1.1. Preparation of the Ligands

The H_2_L^1,2^ ligands were obtained by a condensation reaction of 5-nitrobenzaldehyde (0.4315 g, 2.836 mmol) and 2-hydroxy or 4-hydroxybenzhydrazide (0.4739 g, 2.836 mmol), in a molar ratio of 1:1, in ethanol (30 mL). Suitable crystals of the H_2_L^2^ ligand were obtained, and the molecular and crystal structures were determined.

H_2_L^1^: Color: yellow, Yield: 70.22%
IR-ATR: 3336 cm^−1^ (N–H), 1651 cm^−1^ (C=O), 1603 cm^−1^ (C=N), 1479 cm^−1^ (C=C), 1275 cm^−1^ (C–O)
EA: C_theo_: 55.82, C_found_: 55.56, H_theo_: 3.68, H_found_: 3.57, N_theo_: 13.95, N_found_: 13.81%.
DSC: Onset 302.82 °C, *E* = 2.86 kJ mol^−1^
H_2_L^2^: Color: yellow, Yield: 93.26%
IR-ATR: 3317 cm^−1^ (N–H), 1651 cm^−1^ (C=O), 1588 cm^−1^ (C=N), 1502 cm^−1^ (C=C), 1238 cm^−1^ (C–O)
EA: C_theo_: 55.82, C_found_: 55.70, H_theo_: 3.68, H_found_: 3.49, N_theo_: 13.95, N_found_: 13.63%.
DSC: Onset 304.57 °C, *E*= 0.21 kJ mol^−1^

#### 2.1.2. Preparation of the Complexes

**Mononuclear complexes [MoO_2_(L^1 or 2^)(alcohol)]:** [MoO_2_(acac)_2_] (0.0517 g, 0.11851 mmol) were dissolved in the appropriate alcohol (methanol, ethanol, or 2-propanol), and the appropriate ligand, H_2_L^1^ or H_2_L^2^ (0.0478 g, 0.11851 mmol), was added. The solution was refluxed for 3 h and was left at room temperature. After several days, the product was obtained. The product was filtered off and dried in the desiccator. The samples were analyzed after a prolonged period of standing at room temperature:

**[MoO_2_(L^1^)(MeOH)]:** Color: yellow, Yield: 46.89%
IR-ATR: 1606 cm^−1^ (C=N), 1525 cm^−1^ (C=C), 1267 cm^−1^ (C–O), 1017 cm^−1^ (MeOH), 917 and 892 cm^−1^ (Mo=O)
TGA: MeOH_theo_: 6.98%, MeOH_exp_: 7.45%, MoO_3theo_: 29.25%, MoO_3exp_: 27.37%.
EA: C_theo_: 39.23, C_found_: 39.13, H_theo_: 2.85, H_found_: 2.68, N_theo_: 9.15, N_found_: 9.02%.
**[MoO_2_(L^1^)(EtOH)]:** Color: yellow, Yield: 52.98%
IR-ATR: 1606 cm^−1^ (C=N), 1515 cm^−1^ (C=C), 1270 cm^−1^ (C–O), 1042 cm^−1^ (EtOH), 918 and 903 cm^−1^ (Mo=O)
TGA: EtOH_theo_: 9.75%, EtOH_exp_: 10.3%, MoO_3theo_: 30.49%, MoO_3exp_: 28.51%.
EA: C_theo_: 40.61, C_found_: 40.49, H_theo_: 3.19, H_found_: 3.06, N_theo_: 8.88, N_found_: 8.55%.
**[MoO_2_(L^1^)(2-PrOH)]∙2-PrOH:** Color: yellow, Yield: 24.57%
IR-ATR: 1604 cm^−1^ (C=N), 1503 cm^−1^ (C=C), 1250 cm^−1^ (C–O), 943 cm^−1^ (PrOH), 921 and 909 cm^−1^ (Mo=O)
TGA: PrOH_theo_: 22.01%, PrOH_exp_: 19.6%, MoO_3theo_: 26.34%, MoO_3exp_: 25.26%.
EA: C_theo_: 43.89, C_found_: 43.79, H_theo_: 4.60, H_found_: 4.48, N_theo_: 7.68, N_found_: 7.53%.
**[MoO_2_(L^2^)(MeOH)]:** Color: yellow, Yield: 31.22%
IR-ATR: 1602 cm^−1^ (C=N), 1491 cm^−1^ (C=C), 1269 cm^−1^ (C–O), 1017 cm^−1^ (MeOH), 941 and 907 cm^−1^ (Mo=O)
TGA: MeOH_theo_: 6.98%, MeOH_exp_: 7.21%, MoO_3theo_: 29.25%, MoO_3exp_: 28.46%.
EA: C_theo_: 39.23, C_found_: 39.07, H_theo_: 2.85, H_found_: 2.70, N_theo_: 9.15, N_found_: 9.06%.

**Mononuclear complex [MoO_2_(L^2^)(H_2_O)]:** [MoO_2_(acac)_2_] (0.0524 g, 0.1607 mmol) was dissolved in acetonitrile, ethanol, or 2-propanol (30 mL), and the H_2_L^2^ ligand (0.0484 g, 0.1607 mmol) was added. The solution was refluxed for 2 h and was left at room temperature. The product was filtered off and dried in the desiccator.

Color: yellow, Yield: 76.04%

IR-ATR: 1606 cm^−1^ (C=N), 1505 cm^−1^ (C=C), 1271 cm^−1^ (C–O), 950 and 896 cm^−1^ (Mo=O)

TGA: H_2_O_theo_: 4.03%, H_2_O_exp_: 4.39%, MoO_3theo_: 32.33%, MoO_3exp_: 30.55%.

EA: C_theo_: 37.77, C_found_: 37.65, H_theo_: 2.49, H_found_: 2.36, N_theo_: 9.44, N_found_: 9.17%.

**Polynuclear complex [MoO_2_(L^1^)]*_n_*:** [MoO_2_(acac)_2_] (0.0520 g, 0.15934 mmol) was dissolved in acetonitrile (30 mL), and the H_2_L^1^ ligand (0.0480 g, 0.15934 mmol) was added. The solution was refluxed for 2 h, and a product formed during the reaction. The product was filtered off and dried in the desiccator.

Color: brown, Yield: 38.30%
IR-ATR: 1602 cm^−1^ (C=N), 1513 cm^−1^ (C=C), 1215 cm^−1^ (C–O), 829 and 810 cm^−1^ (Mo=O)
TGA: MoO_3theo_: 33.69%, MoO_3exp_: 32.67%.
EA: C_theo_: 39.36, C_found_: 39.21, H_theo_: 2.12, H_found_: 2.04, N_theo_: 9.84, N_found_: 9.62%.

### 2.2. Stability Investigation

Stability tests were performed with mononuclear complexes [MoO_2_(L^1^)(MeOH)], [[MoO_2_(L^1^)(EtOH)] [MoO_2_(L^1^)(2-PrOH)]∙2-PrOH, MoO_2_(L^2^)(MeOH)], and [MoO_2_(L^2^)(H_2_O)]. The complexes were heated to 290 °C and left at the corresponding temperature for 1 h. After they had cooled to room temperature, further analyses were performed.

### 2.3. Impedance Spectroscopy Measurements

The electrical and dielectric properties of the compounds [MoO_2_(L^1^)(MeOH)], [MoO_2_(L^2^)(MeOH)], [MoO_2_(L^2^)(H_2_O)], and [MoO_2_(L^1^)]*_n_* were studied via impedance spectroscopy (IS). Complex impedance was measured over a wide range of frequencies (0.01 Hz to 1 MHz) and temperatures (30–210 °C, step 30 °C) using an impedance analyzer (Novocontrol Alpha-AN Dielectric Spectrometer, Novocontrol Technologies GmbH & Co. KG, Hundsangen, Germany). The temperature was controlled to ±0.2 °C. The measurements were performed on polycrystalline powder samples pressed into cylindrical pellets with a diameter of 5 mm diameter and a thickness of 1 mm under a uniform load of 2 10^3^ kg using a hydraulic press. For the electrical contact, gold electrodes with a diameter of 3.8 mm were sputtered onto both sides of the pellets using an SC7620 sputter coater from Quorum Technologies (Laughton, UK). The experimental data were analyzed by electrical equivalent circuit (EEC) modeling using the complex nonlinear least-squares (CNLLSQ) fitting procedure and WinFIT software (version 3.2, Novocontrol Technologies GmbH & Co. KG, Hundsangen, Germany).

### 2.4. Physical Methods

Elemental analyses were conducted by the Analytical Services Laboratory of the Ruđer Bošković Institute, Zagreb. Thermogravimetric (TG) analyses were performed using a Mettler TGA/DSC3+ thermobalance in aluminum crucibles. All experiments were performed in an oxygen atmosphere with a flow rate of 200 cm^3^ min^−1^ and with heating rates of 10 K min^−1^. Differential scanning calorimetry (DSC) measurements were carried out using a Mettler-Toledo DSC823e calorimeter and analyzed with Mettler STARe 9.01 software. IR-ATR spectra were recorded on a Perkin Elmer Spectrum Two spectrometer in the spectral range between 4500–450 cm^−1^.

Single-crystal X-ray diffraction: Single crystals of H_2_L^2^, [MoO_2_(L^1^)(H_2_O)], [MoO_2_(L^2^)(H_2_O)], [MoO2(L^1^)]_n_, [MoO_2_(L^1^)(MeOH)]·MeOH, [MoO_2_(L^1^)(EtOH)]·EtOH, and [MoO_2_(L^1^)(2-PrOH)]·(2-PrOH) of suitable quality were selected for the diffraction experiments. Data were collected using a Rigaku XtaLAB Synergy-S diffractometer armed with a Dualflex source (Cu *K*α radiation, *λ* = 1.54184 Å) and a HyPix detector. Data were collected via *ω*-scans at 170 K. Data were processed with the CrysAlis program package [21]. A summary of the general crystal data, along with the data collection and structure refinement parameters, is presented in Table S1 (Supplementary Materials). The structures were solved via dual-space methods using SHELXT [22]. The refinement procedure was accomplished via full-matrix least-squares methods based on *F*^2^ values against all reflections, including the anisotropic displacement parameters for all non-H atoms. Hydrogen atoms bound to carbon atoms were placed in geometrically idealized positions and refined by using the riding model, with *U*_iso_ = 1.2 *U*_eq_ of the connected carbon atom, or as ideal CH_3_ groups, with *U*_iso_ = 1.5 *U*_eq_. Hydrogen atoms attached to heteroatoms were located in the different Fourier maps in the final stages of the refinement procedure. Their coordinates were refined freely but with restrained N−H distances of 0.86 (2) and O–H distances of 0.84 (2) A. All refinements were conducted using SHELXL [23]. The SHELX programs were operated within the Olex2 suite [24]. Geometrical calculations were performed and molecular graphics were produced using Mercury [25]. The structure for H_2_L^2^ was solved as a two-component non-merohedral twin (for details, see the Supplementary Materials), with the two twin domains being present in a ratio of 0.436:0.564. The data for [MoO_2_(L^2^)(H_2_O)] were treated for twinning. The twin law was found to be (−1, 0, 0, 0, −1, 0, 0, 0, 1), while the two twin components were present in the 0.212:0.788 ratio.

## 3. Results and Discussion

### 3.1. Preparation and Characterization of Molybdenum Complexes

Hydrazone ligands were prepared via the reaction of 2-hydroxy-5-nitrobenzaldehyde with 2-hydroxybenzhydrazide or 4-hydroxybenzhydrazide, 1:1 ratio, in methanol. High yields were obtained: 71% for H_2_L^1^ and 93% for H_2_L^2^. DSC analysis gave insight into the ligands’ melting points and purity (Figures S1 and S2). For the H_2_L^1^ ligand, an endothermic peak at 302 °C was attributed to the melting process and was followed by exothermic decomposition. For the H_2_L^2^ ligand, the melting onset occurred at 304 °C. The IR spectra for both ligands are similar; characteristic C=O absorption bands are present at 1651 cm^−1^ for H_2_L^1 and 2^, while bands at 1601 cm^−1^ H_2_L^1^ and 1588 cm^−1^ H_2_L^2^ are characteristic for C=N. C–O_phenyl_ bands are present at 1275 cm^−1^ for H_2_L^1^ and at 1238 cm^−1^ for H_2_L^2^.

Further reactions of the prepared ligands with the molybdenum starting compound [MoO_2_(C_5_H_7_O_2_)_2_] in different solvents provided dioxidomolybdenum(VI) coordination complexes. When H_2_L^1^ reacted with [MoO_2_(C_5_H_7_O_2_)_2_] in methanol, ethanol, or 2-propanol, the mononuclear complex of the general formula [MoO_2_(L^1^)(alcohol)]∙alcohol was obtained. While standing at room temperature, the solvent molecule spontaneously left the crystal structure for the complexes obtained from methanol and ethanol, and the complexes were analyzed as were the [MoO_2_(L^1^)(alcohol)] complexes (Figures S3 and S4). The complex obtained from 2-propanol remained as the crystallized solvent molecule, even after a longer period, which can be clearly seen in the thermogram (Figure S5). On the other hand, the reaction of H_2_L^2^ and [MoO_2_(C_5_H_7_O_2_)_2_] in methanol resulted in the formation of [MoO_2_(L^2^)(MeOH)], but the reactions in ethanol, 2-propanol, and even acetonitrile provided [MoO_2_(L^2^)(H_2_O)]. Upon heating all of the mononuclear compounds, the coordinated solvent molecule loss was recorded in the temperature range of 99–126 °C for [MoO_2_(L^1^)(MeOH)], 117–145 °C for [MoO_2_(L^1^)(EtOH)], 92–135 °C for [MoO_2_(L^1^)(2-PrOH)]∙2-PrOH, 100–160 °C for [MoO_2_(L^2^)(MeOH)], and 170–194 °C for [MoO_2_(L^2^)(H_2_O)]. Further losses at 342–488 °C for [MoO_2_(L^1^)(MeOH)], 345–454 °C for [MoO_2_(L^1^)(EtOH)], 327–444 °C for [MoO_2_(L^1^)(2-PrOH)] ∙2-PrOH, 345–460 °C for [MoO_2_(L^2^)(MeOH)], and 343–460 °C for [MoO_2_(L^2^)(H_2_O)] were attributed to the decomposition of the complexes’ decomposition. The final residue, a white powder, was analyzed by IR and PXRD and compared to commercially available MoO_3_. Surprisingly, via the reaction of the H_2_L^1^ and [MoO_2_(C_5_H_7_O_2_)_2_] in acetonitrile, a brown polynuclear compound [MoO_2_(L^1^)]*_n_* was obtained. TG analysis of the compound [MoO_2_(L^1^)]*_n_* resulted in a one-step thermogram, a loss in the range 356–466 °C attributed to the complex’s decomposition, and the formation of MoO_3_ residue (Figure S6). Further, dark brown compounds were obtained when the yellow mononuclear samples were heated at 280 °C, characterized as polynuclear ones, [MoO_2_(L^1or2^)]*_n_*. Stability tests were also performed by exposing the samples [MoO_2_(L^1^)(MeOH)], [MoO_2_(L^1^)(EtOH)], [MoO_2_(L^1^)(2-PrOH)∙2-PrOH, [MoO_2_(L^2^)(MeOH)], and [MoO_2_(L^2^)(H_2_O)] (in the solid state) to solvent vapors in order to determine whether the coordinated solvent molecule could be replaced by the solvent of the exposed vapors. The samples were exposed to the solvent vapors for 24 h, after which IR spectra of the samples were recorded and compared to the initial sample (Figure S7, up and down). Additionally, the existence of the corresponding compound was confirmed by TGA as well. As expected, the complexes obtained from the H_2_L^1^ ligand could be easily transformed into other mononuclear analogs, while the complexes obtained from H_2_L^2^ were more rigid (Table 1), which also correlates with the results obtained from their solution-based synthesis. An unusual feature was noticed in the complex [MoO_2_(L^1^)(MeOH)] as exposed to EtOH vapors since [MoO_2_(L^1^)(H_2_O)] was formed, which was not in accordance with the results obtained from the solvent-based synthesis. The new complex [MoO_2_(L^1^)(H_2_O)] was analyzed by TGA, which confirmed water coordination (H_2_O_theo_: 4.03%, H_2_O_exp_: 5.09%, MoO_3theo_: 32.33%, MoO_3exp_: 31.93%) and the thermogram was very similar to the one for the complex [MoO_2_(L^2^)(H_2_O)]. Due to heating the sample [MoO_2_(L^1^)(MeOH)], water decoordinated the complex in the range of 98–137 °C, and further complex decomposition occurred in the range of 338–451 °C. Once isolated, all of the samples were very stable and remained unchanged, even after a few months.

In all of the obtained complexes, the ligands were coordinated to the MoO_2_^2+^ core as a doubly deprotonated one, through ONO atoms, which was indicated by the IR spectra as the band at ~1340 cm^−1^, assigned to the C–O group of the hydrazone moiety, and the bands at ~1600 cm^−1^ and 1250 cm^−1^, belonging to C=N_imine_ and C–O_phenyl_ groups. Furthermore, in all of the mononuclear complexes, {MoO_2_}^2+^ core vibrations appeared around ~920 and ~900 cm^−1^. In the spectra of [MoO_2_(L^1or 2^)(MeOH)], a new band appeared at ~1017 cm^−1^ due to the C–O_MeOH_ vibrations in the spectra of the [MoO_2_(L^1^)(EtOH)] band at 1042 cm^−1^, which is characteristic for C–O_EtOH_ vibrations, and in the spectra of [MoO_2_(L^1^)(PrOH)]∙2-PrOH band at 943 cm ^−1^, which is characteristic for C–O_PrOH_ vibrations. In the polynuclear complexes [MoO2(L^1^)]_n_, polymerization occurred through the terminal O atom, assigned to the bands at 829 and 810 cm^−1^ in the spectra [26,27].

### 3.2. Description of Molecular and Crystal Structures

The molecular and crystal structures of the H_2_L^2^ ligand and the complexes [MoO_2_(L^1^)(H_2_O)], [MoO_2_(L^2^)(H_2_O)], [MoO2(L^1^)]_n_, [MoO_2_(L^1^)(MeOH)]·MeOH, [MoO_2_(L^1^)(EtOH)]·EtOH, and [MoO_2_(L^1^)(2-PrOH)]·2-PrOH, as described in this work, were elucidated via single-crystal X-ray diffraction (SC-XRD) experiments. Basic crystallographic data and refinement parameters are given in the Supplementary Materials in Table S1. A detailed description of the molecular and crystal structure of H_2_L^2^ is given in the Supplementary Materials (Figures S8 and S9 and Table S2). The suitable single crystals of the H_2_L^2^ ligand and the complexes [MoO_2_(L^2^)(H_2_O)], [MoO2(L^1^)]_n_, [MoO_2_(L^1^)(MeOH)]·MeOH, [MoO_2_(L^1^)(EtOH)]·EtOH, and [MoO_2_(L^1^)(2-PrOH)]·2-PrOH were obtained via the conventional solution synthesis, while the single crystals of [MoO_2_(L^1^)(H_2_O)] were obtained via the solvothermal reaction of the H_2_L^1^ ligand, [MoO_2_(acac)_2_] in methanol.

**Figure 1:**
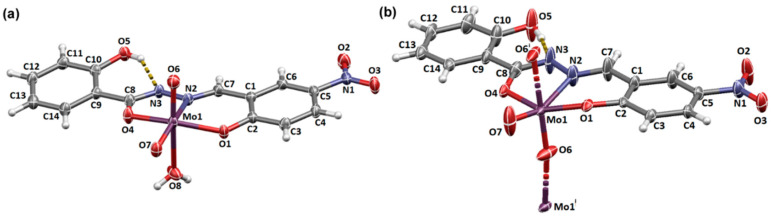
Molecular structures with the atom-labeling scheme of: (**a**) [MoO_2_(L^1^)(H_2_O)]; and (**b**) the repeating unit in [MoO2(L^1^)]_n_. The displacement ellipsoids are drawn at a 50% probability level for (**a**) and at a 30% probability level for (**b**) at 170 K. Hydrogen atoms are presented as spheres of arbitrary, small radii. The intramolecular hydrogen bonds of the O–H···N type are highlighted as yellow dashed lines. In (**b**), the symmetry code i = 1/2+*x*,*y*,3/2-*z*.

**Figure 2:**
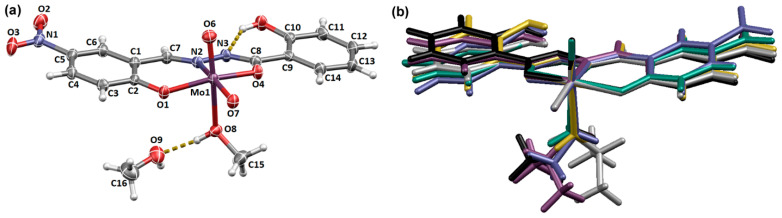
(**a**) Molecular structure with the atom-labeling scheme for [MoO_2_(L^1^)(MeOH)]·MeOH. The displacement ellipsoids are drawn at a 50% probability level at 170 K. Hydrogen atoms are presented as spheres of arbitrary, small radii. The intramolecular hydrogen bonds of the O–H···N type are highlighted as yellow dashed lines; (**b**) Overlay of the coordination entities: [MoO_2_(L^1^)(H_2_O)] (yellow), [MoO_2_(L^2^)(H_2_O)] (green), [MoO_2_(L^1^)(MeOH)] (blue), [MoO_2_(L^1^)(EtOH)] (black), and [MoO_2_(L^1^)(2-PrOH)] (molecule A, gray; molecule B, purple).

In all of the examined complexes, the ligand (H_2_L^1^ or H_2_L^2^) is coordinated in its doubly deprotonated form of the imino tautomer [28,29], thus affording a tridentate *ONO* coordination mode (Figure S10, Table S4). Coordination geometry around molybdenum atoms in all of the complexes can be described as a distorted octahedral one, being fulfilled by the *cis*-arranged oxo ligands, and, except for [MoO2(L^1^)]_n_ (Figure 1a), an ancillary water or alcohol molecule. In [MoO2(L^1^)]*_n_*, the octahedral environment of each Mo atom is realized through the Mo=O···Mo interaction with the neighboring [MoO2(L^1^)] unit (Figure 1b). This is consistent with the tendency of *cis*-dioxomolybdenum(VI) complexes to form polynuclear assemblies in the absence of a suitable donor for the sixth coordination site [30]. The longest distances within the Mo coordination sphere are those positioned *trans* to the oxo ligands, i.e., the Mo1–N2 and Mo1–O6 bonds (Mo1A–N2A, Mo1A–O6A, and Mo1B–N2B; Mo1B–O6B in [MoO_2_(L^1^)(2-PrOH)]·(2-PrOH). The main geometrical features of the Mo(VI) complexes investigated here are comparable with those of Mo(VI) complexes with similar ligands reported in the literature [6,7,8,31,32]. The largest differences in the molecular geometries between the complexes can be observed in the relative position of the phenyl rings of the aldehyde and hydrazone residues (Figure 1b and Table S5, Figure 2). It is also interesting to observe that, in all of the inspected complexes, the Mo atom is shifted from the plane defined by the coordinating O1, N2, O4, and O7 atoms toward the oxo O6 atom (Table S5).

In the structures of [MoO_2_(L^1^)(H_2_O)] and [MoO_2_(L^2^)(H_2_O)], the coordination entities associate via O–H···O, N–H···O, and C–H···O hydrogen bonds. However, the resulting pattern is more complex in the case of [MoO_2_(L^2^)(H_2_O)], where the O5–H5 moiety is disposable for intermolecular hydrogen bonding, which is not the case in [MoO_2_(L^1^)(H_2_O)] (Table S6 and Figures S11 and S12). Crystal packing in the [MoO2(L^1^)]_n_ relies exclusively on C–H···O interactions since the only good hydrogen-bond donor, O5–H5 is functionally engaged in the intramolecular hydrogen bonding (Table S6 and Figure S13). In the structures of Mo(VI) complexes with alcohols as ancillary ligands, which crystalize as solvates, the presence of additional solvent molecules leads to an exceedingly rich pattern of intermolecular interactions (Table S6 and Figures S14 and S15).

### 3.3. Impedance Spectroscopy Investigation of the Chosen Molybdenum Complexes

For the impedance spectroscopy study, we selected four samples prepared from methanol and acetonitrile. The complexes prepared from methanol provided mononuclear compounds [MoO_2_(L^1or2^)(MeOH)], while the ones prepared from acetonitrile provided either a polynuclear compound [MoO_2_(L^1^)]*_n_* or a mononuclear compound [MoO_2_(L^2^)(H_2_O)], depending on the used ligand. Based on the results of the thermal behavior, [MoO_2_(L^1^)(MeOH)], [MoO_2_(L^2^)(MeOH)], [MoO_2_(L^2^)(H_2_O)], and [MoO_2_(L^1^)]*_n_* complexes were heated from 30 to 230 °C, after which they were cooled to 30 °C. The temperature of 230 °C was chosen based on the TG analysis since there is no coordinated solvent molecule left in the crystal structure above this temperature and the obtained compounds are stable up to ~300 °C.

**Figure 3:**
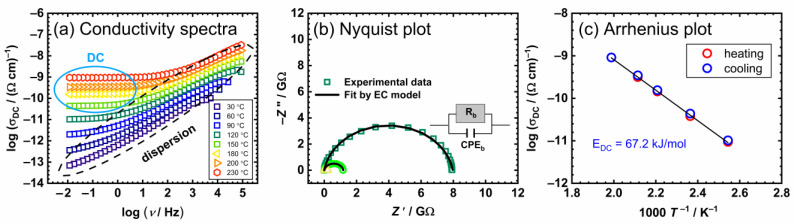
(**a**) Conductivity spectra; (**b**) Nyquist plot; and (**c**) Arrhenius plot—temperature dependence of DC conductivity (log(*σ*_DC_) vs. 1000/T) for compound [MoO_2_(L^1^)]*_n_*. In (**c**), the heating run is marked with red circles, and cooling is marked with blue circles.

The frequency dependence of the real part of the electrical conductivity, *σ′*, or the conductivity spectra at different temperatures for the sample [MoO_2_(L^1^)]*_n_*, is presented in Figure 3a. It is evident that the electrical conductivity, *σ′*, increases with increasing temperature. As for all other samples, the conductivity is thermally activated and shows semiconducting behavior. Moreover, at high temperatures and low frequencies, the conductivity shows no frequency-dependence (constant value), with the so-called plateau corresponding to the DC conductivity (see Figure 3a). In the high-frequency region, the conductivity depends on the frequency and is related to the AC conductivity (dispersion part). Moving from DC to the dispersion region, the crossover shifts to higher frequencies as the temperature increases. Similar behavior in the conductivity spectra can be observed via IS in a wide range of semiconductive materials, amorphous and/or crystalline [33,34,35,36,37,38,39,40,41,42].

Due to the low conductivity of the samples studied, it is obvious that, at lower temperatures, it is not possible to determine the DC value from the graph visually. In this case, the DC conductivity value was obtained by analyzing the experimental complex impedance spectra via EEC modeling using the CNLL S method. (See Figure 3b and the Section 2 for details. The experimental complex impedance spectrum of [MoO_2_(L^1^)]*_n_* was plotted in the complex plane, i.e., with the imaginary part as a function of the real part, Z″ vs. Z′ (a so-called Nyquist diagram). The impedance spectrum with the shape of a semicircle was analyzed by the EEC model, and a good overlap was observed, indicating a good agreement between the experimental data (red curve) and the theoretical model. The EEC model used is based on the R-CPE parallel circuit, where *R* denotes the resistor corresponding to the sample resistance, and the constant phase element, *CPE,* determines the sample capacitance. From the intersection with the X-axis, resistance is calculated @ each temperature, and for the [MoO_2_(L^1^)]_n_ compound @ 200 °C, *R* is equal to 1.06 × 10^9^ Ω. DC conductivity was calculated to be 2.34 × 10^−10^ (Ωcm)^−1^ @ 200 °C. Considering that the sample [MoO_2_(L^1^)]*_n_* is a polynuclear compound, the in situ IS method confirmed that there was no change during the heating/cooling run, which is consistent with the TG analysis and the observed one-step decomposition.

For the studied compound [MoO_2_(L^1^)]*_n_* (see Figure 3a,b), the DC conductivity exhibited an Arrhenius temperature dependence and hence had characteristic activation energy (Figure 3c). The activation energy for DC conductivity, *E*_DC_, was determined from the slope of log(σ_DC_) vs. 1000/T using the following equation: *σ*_DC_ = *σ^∗^*_0_ *e*^[−*E*_DC_/*k*_B_*T*]^, where *σ*_DC_ is the DC conductivity, *σ^∗^*_0_ is the pre-exponential factor, *E*_DC_ is the activation energy, k_B_ is the Boltzmann constant, and *T* is the temperature (K). It can be seen that there are insignificant differences between the heating and the cooling runs, which is another indicator that, in the specified temperature range, there was no change and/or possible transformation of the compound [MoO_2_(L^1^)]*_n_*. This is to be expected since this compound is stable and does not contain molecules of coordinated or crystalline solvents that would be lost during heating. The low activation energy (67.2 kJ mol^−1^) of this type of compound indicates that the polynuclear complex, in which the polymerization was achieved through the terminal oxygen atom, enabled a significant and uninterrupted transfer of charge carriers through this structure.

**Figure 4:**
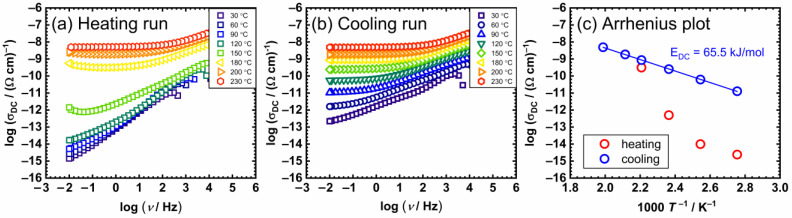
Conductivity spectra for [MoO_2_(L^1^)(MeOH)] in heating (**a**) and cooling (**b**) runs, and (**c**) temperature dependence of DC conductivity (log(σ_DC_) vs. 1000/T) for both runs (red circle—heating, blue circle—cooling).

On the other hand, for samples [MoO_2_(L^1^)(MeOH)], [MoO_2_(L^2^)(MeOH)], and [MoO_2_(L^2^)(H_2_O)], there was a certain change. Figure 4 shows the conductivity spectra during the heating and cooling cycles for the sample [MoO_2_(L^1^)(MeOH)], the thermogram for which shows a two-step decomposition, as discussed above. As previously stated, the first step corresponded to the release of the coordinated methanol molecule which, according to the TG curve, took place in the range of 100–126 °C.

Structural change associated with the release of a coordinated methanol molecule can be seen in situ in the conductivity spectra, as shown in Figure 4. During the heating run, a non-monotonic change with the increase in temperature was visible, i.e., a sudden jump in conductivity above 120 °C, with emphasis on the temperature range between 120–180 °C (see Figure 4a), corresponding to the first stage of decay visible in the TG curve. In contrast, during the cooling run, a monotonic change of conductivity with temperature was found (see Figure 4b). It can be assumed that the compound of the mononuclear complex polymerized after heating to 230 °C as it lost solvent (methanol), which was also confirmed by IR spectroscopy. The compounds [MoO_2_(L^2^)(MeOH)] and [MoO_2_(L^2^)(H_2_O)] showed similar behavior along with non-monotonic changes in conductivity with increase in temperature in the heating cycle, which could be related to structural change/transformation (see Figure S17). These three mononuclear compounds, in contrast to [MoO_2_(L^1^)]*_n_*, show a linear temperature dependence (1000/T) of DC conductivity only in the cooling run. An example of a [MoO_2_(L^1^)(MeOH)] compound in a heating/cooling cycle is shown in Figure 4c, from which the activation energy was determined for the cooling cycle. The calculated values are listed in Table 2.

According to the results shown in Table 2, it can be assumed that the [MoO_2_(L^1^)(MeOH)] and [MoO_2_(L^2^)(MeOH)] compounds polymerized after heating, which is indicated by a similar value of calculated activation energy, as it was for the [MoO_2_(L^1^)]*_n_* (67.2 vs 65–66 kJ mol^−1^). Although the polymerization of the compound [MoO_2_(L_2_)(H_2_O)] was proven, there was a difference in the activation energy from the methanol-coordinated complexes. This observation can be correlated to the stability of such a compound. Based on the TG study, it is obvious that a longer period of time is needed for polymerization in the case of the water-coordinated compound, and as result, this reflects upon the electrical properties and the activation energy as well. Moreover, we should emphasize here the interesting behavior observed for the sample [MoO_2_(L^2^)(MeOH)], which showed low activation energy. However, its conductivity was still several orders of magnitude lower compared to similar compounds, where polymerization is expected upon heating, including [MoO_2_(L^1^)(MeOH)] and the polymer [MoO_2_(L^1^)]*_n_*. This effect should be further investigated.

**Figure 5:**
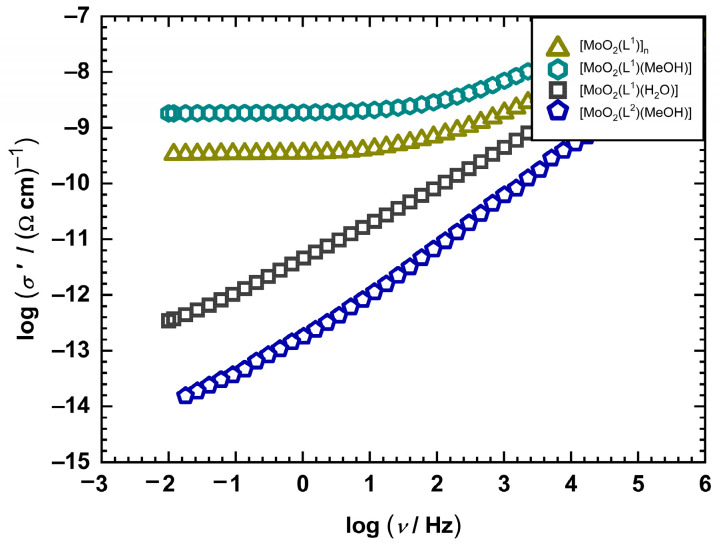
Conductivity isotherms for the studied compounds obtained @200 °C.

Furthermore, the conductivity isotherms @200 °C in the cooling run for all of the studied compounds are shown in Figure 5, and the corresponding DC conductivity values are shown in Table 3. 

Based on Table 3**,** the following conductivity trend can be seen in the cooling cycle @200 °C: *σ* ([MoO_2_(L^1^)(MeOH)]) > *σ* ([MoO_2_(L^1^)]*_n_*) > *σ* ([MoO_2_(L^2^)H_2_O)] > *σ* ([MoO_2_(L^2^)(MeOH)]). Here, a few comments are in order. First, the compound [MoO_2_(L^2^)(H_2_O)], obtained from the H_2_L^2^ ligand, shows significantly lower conductivity in comparison to [MoO_2_(L^1^)(MeOH)] and ([MoO_2_(L^1^)]*_n_*). This fact could be attributed to the type of ligand and the slow release of the coordinated solvent during heating. Accordingly, its activation energy was significantly higher, in the range of 100 kJ mol^–1^. Second, the structural change (polymerization) after the solvent release during heating for compounds [MoO_2_(L^1^)(MeOH)], ([MoO_2_(L^2^)(MeOH)], and [MoO_2_(L^2^)(H_2_O)] was indicated by the observed differences in the conductivity spectra in the heating vs. the cooling runs (see Figure 4). It can be observed that, at lower temperatures for all compounds, the conductivity was almost temperature-independent, and the increase in conductivity was visible only above 120 °C. Furthermore, the [MoO_2_(L^1^)(MeOH)] sample polymerized and had higher conductivity in the cooling run as compared to the heating run (see Figure 5). On the other hand, in the [MoO_2_(L^2^)(MeOH)] and [MoO_2_(L^2^)(H_2_O)] compounds (see Figure S17), the release of the solvent molecule (crystalline, coordinated) was observed at higher temperatures, and the conductivity in the cooling run was somewhat lower, with a difference of almost an order of magnitude in the case of the complex [MoO_2_(L^2^)(H_2_O)]. Finally, an interesting, but still unexpected, behavior for the compound [MoO_2_(L^2^)(MeOH)] should be emphasized. Although the complex showed a low activation energy, its DC conductivity was still several orders of magnitude lower compared to the compounds prepared by reactions with the H_2_L^1^ ligand, namely: [MoO_2_(L^1^)(MeOH)] and [MoO_2_(L^1^)]_n_. However, the activation energy value (66.0 kJ/mol) for the sample [MoO_2_(L^1^)(MeOH)] indicated polymerization, while differences in the conductivity values did not. It should certainly be taken into account the type of H_2_L^2^ ligand, and the observed steric influence of the ligand on conductivity transport should not be ruled out in compounds [MoO_2_(L^2^)(H_2_O) and [MoO_2_(L^2^)(MeOH)]. As mentioned previously, this behavior needs to be investigated in more detail.

## 4. Conclusions

New molybdenum complexes containing hydrazine ligands obtained from 5-nitrobenzaldehyde and 2-hydroxy or 4-hydroxybenzhydrazide were prepared and characterized. Chosen Mo(VI) compounds were investigated by the impedance spectroscopy method. For the first time, impedance spectroscopy was used to follow in situ transformations of mononuclear to polynuclear complexes, supporting the results obtained by heat treatment and thermogravimetric analysis for mononuclear compounds. All of the studied complexes showed an increase in conductivity with temperature; in other words, they behaved as semiconductors. Samples with high conductivity certainly open the possibility for the application of such complexes as semiconductors in various devices. For instance, the sample [MoO_2_(L^1^)]*_n_*, along with [MoO_2_(L^1^)(MeOH)], the conductivity of which increased by four orders of magnitude after the heating cycle and structural transformation, turned out to be potential candidates for target application due to the considerably high conductivity that they exhibited for this class of materials. Future research will be directed in that direction, along with the preparation of analogous complexes with different transition metal centers (i.e., Cu or V) in order to correlate the obtained electric results.

## Figures and Tables

**Table 1:** Stability tests with different mononuclear complexes.

Mo Complex	MeOH Vapors	EtOH Vapors	2-PrOH Vapors
[MoO_2_(L^1^)(MeOH)]	-	[MoO_2_(L^1^)(H_2_O)]	[MoO_2_(L^1^)(2-PrOH)]∙2-PrOH
[MoO_2_(L^1^)(EtOH)]	[MoO_2_(L^1^)(MeOH)]	-	[MoO_2_(L^1^)(2-PrOH)]∙2-PrOH
[MoO_2_(L^1^)(2-PrOH)]∙2-PrOH	[MoO_2_(L^1^)(MeOH)]	[MoO_2_(L^1^)(EtOH)]	-
[MoO_2_(L^2^)(MeOH)]	-	[MoO_2_(L^2^)(H_2_O)]	[MoO_2_(L^2^)(H_2_O)]
[MoO_2_(L^2^)(H_2_O)]	[MoO_2_(L^2^)(MeOH)]	[MoO_2_(L^2^)(H_2_O)]	[MoO_2_(L^2^)(H_2_O)]

**Table 2:** Activation energy values for the DC conductivity in the cooling cycle of the prepared complexes with observed structural changes.

Compounds	*E*_DC_/kJ mol^–1^ (Cooling Run)
[MoO_2_(L^1^)(MeOH)]	65.5
[MoO_2_(L^2^)(MeOH)]	66.0
[MoO_2_(L^2^)(H_2_O)]	105.1

**Table 3:** DC conductivity values for all compounds measured @200 °C in the cooling run.

Compounds	σ_DC_/(Ωcm)^−1^
[MoO_2_(L^1^)(MeOH)]	1.82 × 10^–9^
[MoO_2_(L^2^)(MeOH)]	1.52 × 10^–14^
[MoO_2_(L^2^)(H_2_O)]	3.43 × 10^–13^
[MoO_2_(L^1^)]*_n_*	3.35 × 10^–10^

## Data Availability

Crystallographic data sets for the structures H_2_L^2^, [MoO_2_(L^1^)(H_2_O)], [MoO_2_(L^2^)(H_2_O)], [MoO2(L^1^)]_n_, [MoO_2_(L^1^)(MeOH)]·MeOH, [MoO_2_(L^1^)(EtOH)]·EtOH, and [MoO_2_(L^1^)(2-PrOH)]·2-PrOH are available through the Cambridge Structural Database, with deposition numbers CCDC 2225226 and 2225228-2225233. These data can be obtained free of charge via https://www.ccdc.cam.ac.uk/structures/, accessed on 15 December 2022.

## Supplementary Materials

The following supporting information can be downloaded at: https://www.mdpi.com/article/10.3390/ma16031064/s1, Figure S1. DSC curve for the H_2_L^1^ ligand; Figure S2. TG-DSC curve for the H_2_L^1^ ligand; Figure S3. TG-DSC curve for the mononuclear compound [MoO_2_(L^1^)(MeOH)]; Figure S4. TG-DSC curve for the mononuclear compound [MoO_2_(L^1^)(EtOH)]; Figure S5. TG-DSC curve for the mononuclear compound [MoO_2_(L^1^)(2-PrOH)]∙2-PrOH; Figure S6. TG-DSC curve for the polynuclear compound [MoO_2_(L^1^)]_n_; Figure S7. IR spectra comparison for [MoO_2_(L^1^)(MeOH)] (black), [MoO_2_(L^1^)(EtOH)] (red), and [MoO_2_(L^1^)(2-PrOH)]∙2-PrOH (green); Table S1. General and crystal data, a summary of intensity data collection, and structure refinement for H_2_L^2^, [MoO_2_(L^1^)(H_2_O)], [MoO_2_(L^2^)(H_2_O)], [MoO_2_(L^1^)]_n_, [MoO_2_(L^1^)(MeOH)]·MeOH, [MoO_2_(L^1^)(EtOH)]·EtOH, and [MoO_2_(L^1^)(2-PrOH)]·(2-PrOH); Figure S8. Molecular structures with the atom-labeling scheme for H_2_L^2^; Table S2. Selected geometrical parameters (bond lengths and angles) for H_2_L^2^; Figure S9. Crystal packing in H_2_L^2^ is shown down the: (**a**) a-axis and (**b**) b-axis; Table S3. The geometry of hydrogen bonds and C (Å, o) for H_2_L^2^; Figure S10. Molecular structures with the atom-labeling scheme for: (**a**) [MoO_2_(L^2^)(H_2_O)], (**b**) [MoO_2_(L^1^)(EtOH)]·EtOH, and (**c**) [MoO_2_(L^1^)(2-PrOH)]·2-PrOH; Table S4. Selected geometrical parameters (bond lengths and angles) for [MoO_2_(L^1^)(H_2_O)], [MoO_2_(L^2^)(H_2_O)], [MoO_2_(L^1^)]_n_, [MoO_2_(L^1^)(MeOH)]·MeOH, [MoO_2_(L^1^)(EtOH)]·EtOH, and [MoO_2_(L^1^)(2-PrOH)]·2-PrOH; Table S5. Selected interplanar angles (in °) for [MoO_2_(L^1^)(H_2_O)], [MoO_2_(L^2^)(H_2_O)], [MoO_2_(L^1^)]_n_, [MoO_2_(L^1^)(MeOH)]·MeOH, [MoO_2_(L^1^)(EtOH)]·EtOH, and [MoO_2_(L^1^)(2-PrOH)]·2-PrOH; Table S6. The geometry of hydrogen bonds and C (Å, o) for [MoO_2_(L^1^)(H_2_O)], [MoO_2_(L^2^)(H_2_O)], [MoO_2_(L^1^)]_n_, [MoO_2_(L^1^)(MeOH)]·MeOH, [MoO_2_(L^1^)(EtOH)]·EtOH, and [MoO_2_(L^1^)(2-PrOH)]·2-PrOH; Figure S11. Crystal packing in [MoO_2_(L^1^)(H_2_O)] shown down the: (**a**) a-axis and (**b**) b-axis. Figure S12. Crystal packing in [MoO_2_(L^2^)(H_2_O)] shown down the: (**a**) a-axis and (**b**) b-axis; Figure S13. Crystal packing in [MoO_2_(L^1^)]_n_; Figure S14. Crystal packing in MoO_2_(L^1^)(MeOH)]·MeOH shown down the: (**a**) a-axis and (**b**) c-axis; Figure S15. Crystal packing in MoO_2_(L^1^)(EtOH)]·EtOH shown down the: (**a**) b-axis and (**b**) c-axis; Figure S16. Crystal packing in [MoO_2_(L^1^)(2-PrOH)]·2-PrOH shown down the: (**a**) a-axis and (**b**) c-axis. Figure S17. Conductivity spectra for (a–b) [MoO_2_(L^2^)(MeOH)] and (c–d) [MoO_2_(L^2^)(H_2_O)] in heating (**a**,**c**) and cooling (**b**,**d**) runs.
