# Supplementary material for: Impedance Spectroscopy as a Powerful Tool for Researching Molybdenum-Based Materials with Schiff Base Hydrazones
Source: Materials (Basel). 2023 Jan 25;16(3):1064. doi: 10.3390/ma16031064 (PMC9919434; doi:10.3390/ma16031064)
Supplement: Supplementary file 1 [file materials-16-01064-s001.zip › materials-2129715-supplementary.pdf]

Impedance Spectroscopy as a Powerful Tool for Researching Molybdenum-Based Materials with Schiff Base Hydrazones

Josipa Sarjanović [1], Martina Stojić [1], Mirta Rubčić [1], Luka Pavić [2*] and Jana Pisk [1*]

Contents

Figure S1. DSC curve for the ligand H_2L^1. ... 3
Figure S2. TG-DSC curve for the ligand H_2L^1. ... 3
Figure S3. TG-DSC curve for the mononuclear compound $[MoO_2(L^1)(MeOH)]$. ... 4
Figure S4. TG-DSC curve for the mononuclear compound $[MoO_2(L^1)(EtOH)]$. ... 4
Figure S5. TG-DSC curve for the mononuclear compound $[MoO_2(L^1)(2\text{-}PrOH)]\cdot 2\text{-}PrOH$. ... 5
Figure S6. TG-DSC curve for the polynuclear compound $[MoO_2(L^1)]_n$. ... 5
Figure S7. IR spectra comparison for $[MoO_2(L^1)(MeOH)]$ (black), $[MoO_2(L^1)(EtOH)]$ (red) and $[MoO_2(L^1)(2\text{-}PrOH)]\cdot 2\text{-}PrOH$ (green). ... 6
Table S1. General and crystal data, summary of intensity data collection and structure refinement for H_2L^2, $[MoO_2(L^1)(H_2O)]$, $[MoO_2(L^2)(H_2O)]$, $[MoO2(L^1)]_n$, $[MoO_2(L^1)(MeOH)]\cdot MeOH$, $[MoO_2(L^1)(EtOH)]\cdot EtOH$, $[MoO_2(L^1)(2\text{-}PrOH)]\cdot 2\text{-}PrOH$ ... 7
Description of molecular and crystal structure of H_2L^2 ... 9
Figure S8. Molecular structures with the atom labeling scheme for H_2L^2. The angle between the LS planes defined by C1-C2-C3-C4-C5-C6 ring and O1-N2-O4 fragment is 5.28(7) °. The angle between the LS planes defined by C1-C2-C3-C4-C5-C6 and C9-C10-C11-C12-C13-C14 rings is 22.83(7) °. ... 9
Table S2. Selected geometrical parameters (bond lengths and angles) for H_2L^2 ... 9
Figure S9. Crystal packing in H_2L^2 shown down the: (a) a-axis; (b) b-axis. O–H⋯O and N–H⋯O hydrogen bonds are presented as yellow dashed lines, while C–H⋯O interactions are shown as black dashed lines. ... 11
Table S3. Geometry of hydrogen bonds and C (Å, °) for H_2L^2 ... 12
Description of molecular and crystal structures of Mo(VI) complexes ... 13
Figure S10. Molecular structures with the atom labeling scheme for: (a) $[MoO_2(L^2)(H_2O)]$; (b) $[MoO_2(L^1)(EtOH)]\cdot EtOH$; and (c) $[MoO_2(L^1)(2\text{-}PrOH)]\cdot 2\text{-}PrOH$. The displacement ellipsoids are drawn at 50% probability level at 170 K. Hydrogen atoms are presented as spheres of arbitrary small radii. Intramolecular hydrogen bond of the O–H⋯N type are highlighted by orange dashed line. In (b) only major component of the disordered coordinated ethanol molecule is shown for clarity (the ratio of the occupancies for the two components is ≈ 0.63:0.37). In contrast to remaining structures of Mo(VI) complexes discussed here, the asymmetric unit of $[MoO_2(L^1)(2\text{-}PrOH)]\cdot 2\text{-}PrOH$ contains two coordination moieties and two propanol molecules. For clarity, the molecules in (c) are not shown at their true position within the asymmetric unit. ... 13
Table S4. Selected geometrical parameters (bond lengths and angles) for $[MoO_2(L^1)(H_2O)]$, $[MoO_2(L^2)(H_2O)]$, $[MoO2(L^1)]_n$, $[MoO_2(L^1)(MeOH)]\cdot MeOH$, $[MoO_2(L^1)(EtOH)]\cdot EtOH$, $[MoO_2(L^1)(2\text{-}PrOH)]\cdot 2\text{-}PrOH$ ... 14
Table S5. Selected interplanar angles (in °) for $MoO_2(L^1)(H_2O)]$, $[MoO_2(L^2)(H_2O)]$, $[MoO2(L^1)]_n$, $[MoO_2(L^1)(MeOH)]\cdot MeOH$, $[MoO_2(L^1)(EtOH)]\cdot EtOH$, $[MoO_2(L^1)(2\text{-}$

PrOH)]·(2-PrOH). I represents LS plane passing through the aldehyde subunit (defined by the C1-C2-C3-C4-C5-C6 atoms), while II represents LS plane passing through the aryl ring subunit of the hydrazone part of the molecule (defined by the C9-C10-C11-C12-C13-C14 atoms). L1 represents plane passing through O1, N2 and O4 (chelating atoms). Atoms are numerated according to Figures 1 and 2 and Figure S3.. 21
Table S6. Geometry of hydrogen bonds and C (Å, º) for $[MoO_2(L^1)(H_2O)]$, $[MoO_2(L^2)(H_2O)]$, $[MoO2(L^1)]_n$, $[MoO_2(L^1)(MeOH)]$·MeOH, $[MoO_2(L^1)(EtOH)]$·EtOH, $[MoO_2(L^1)$(2-PrOH)]·2-PrOH.. 22
Figure S11. Crystal packing in $[MoO_2(L^1)(H_2O)]$ shown down the: (a) a-axis; (b) b-axis. O–H···O and N–H···O hydrogen bonds are presented as yellow dashed lines........................... 24
Figure S12. Crystal packing in $[MoO_2(L^2)(H_2O)]$ shown down the: (a) a-axis; (b) b-axis. O–H···O and N–H···O hydrogen bonds are shown as yellow dashed lines. 25
Figure S13. Crystal packing in $[MoO_2(L^1)]_n$. N–H···O hydrogen bonds are presented as yellow dashed lines, while the C–H···O interactions are shown as black dashed lines. 26
Figure S14. Crystal packing in $MoO_2(L^1)(MeOH)]$·MeOH shown down the: (a) a-axis; (b) c-axis. N–H···O and O–H···O hydrogen bonds are presented as yellow dashed lines. 27
Figure S15. Crystal packing in $MoO_2(L^1)(EtOH)]$·EtOH shown down the: (a) b-axis; (b) c-axis. N–H···O and O–H···O hydrogen bonds are presented as yellow dashed lines. 28
Figure S16. Crystal packing in $[MoO_2(L^1)$(2-PrOH)]·2-PrOH shown down the: (a) a-axis; (b) c-axis. N–H···O and O–H···O hydrogen bonds are presented as yellow dashed lines. 29
Figure S17. Conductivity spectra for (a-b) $[MoO_2(L^2)(MeOH)]$ and (c-d) $[MoO_2(L^2)(H_2O)]$ in heating (a,c) and cooling (b,d) run. 30

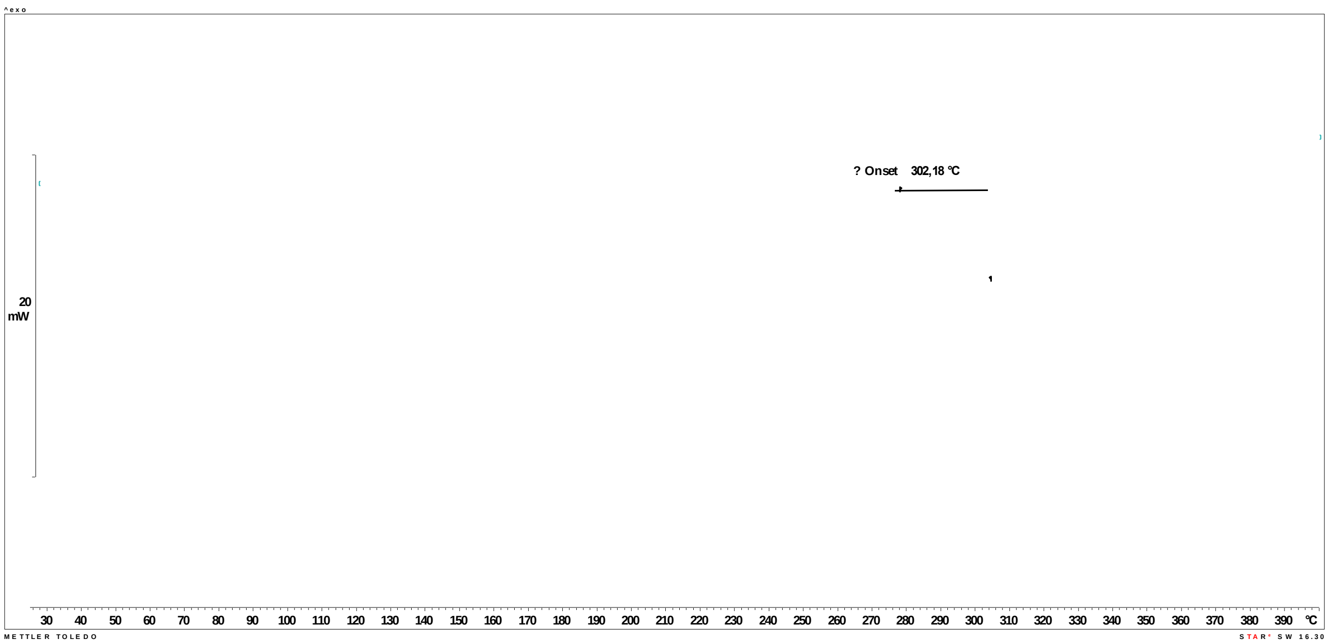

Figure S1. DSC curve for the ligand H_2L^1.

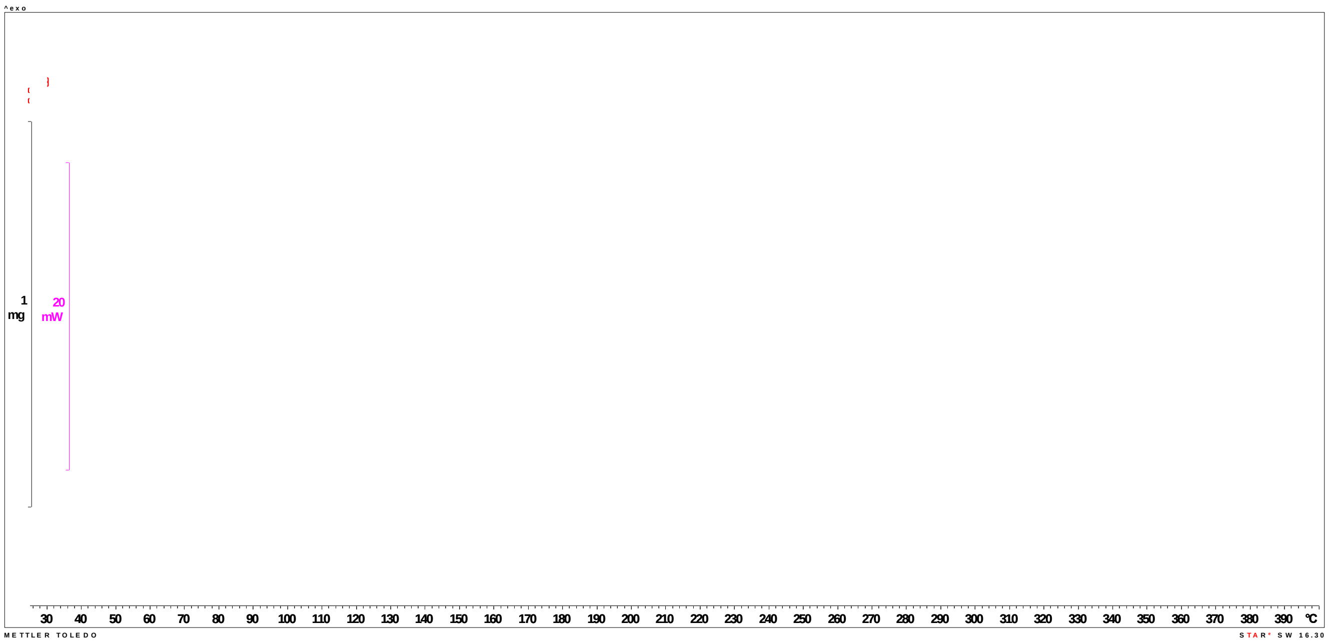

Figure S2. TG-DSC curve for the ligand H_2L^1. Black curve presents weight loss, and pink curve presents heat flow.

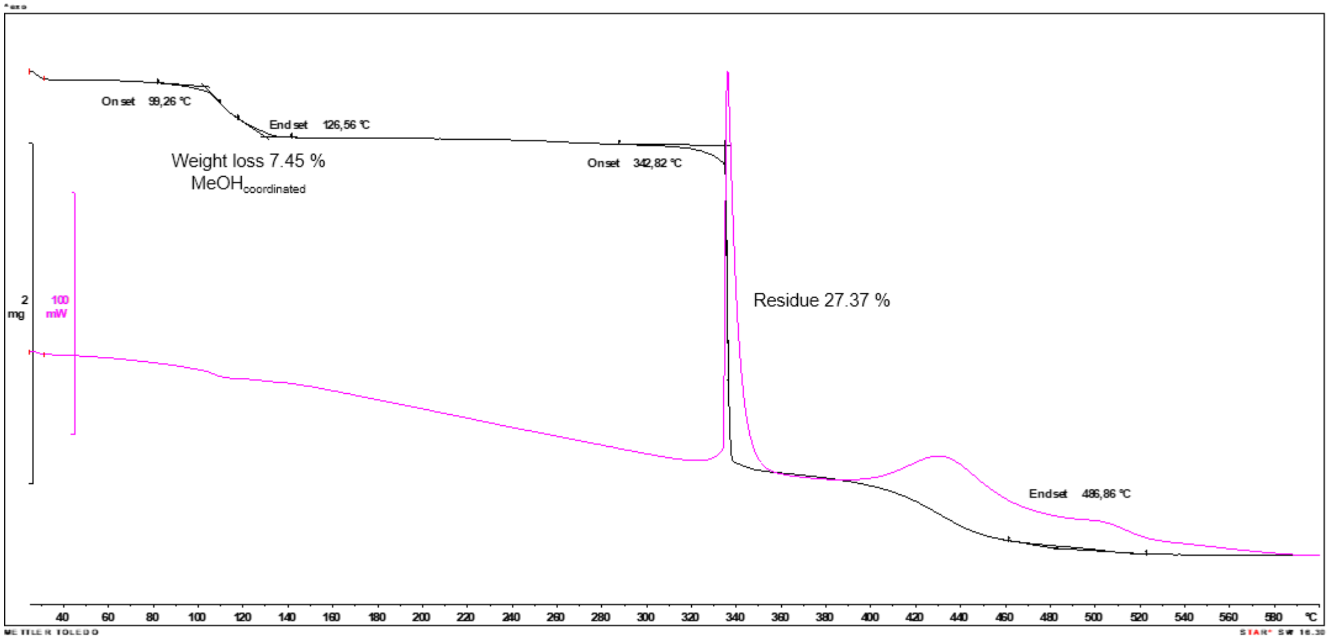

Figure S3. TG-DSC curve for the mononuclear compound [$MoO_2(L^1)(MeOH)$]. Black curve presents weight loss, and pink curve presents heat flow.

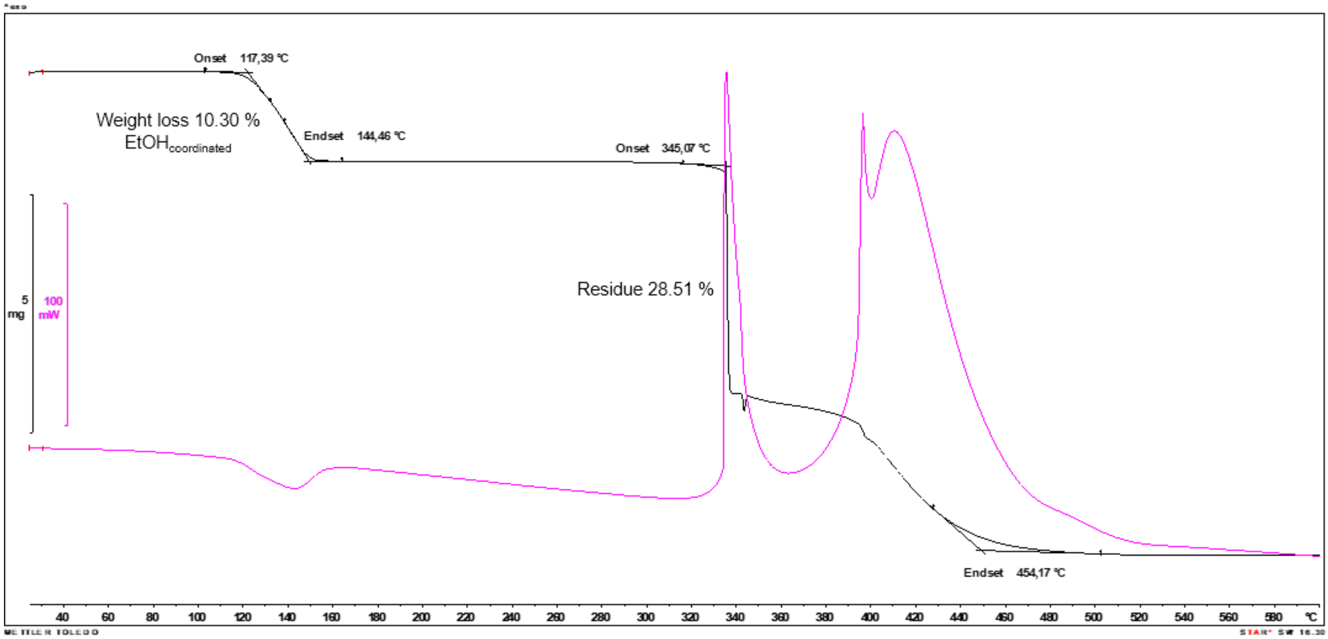

Figure S4. TG-DSC curve for the mononuclear compound [$MoO_2(L^1)(EtOH)$]. Black curve presents weight loss, and pink curve presents heat flow.

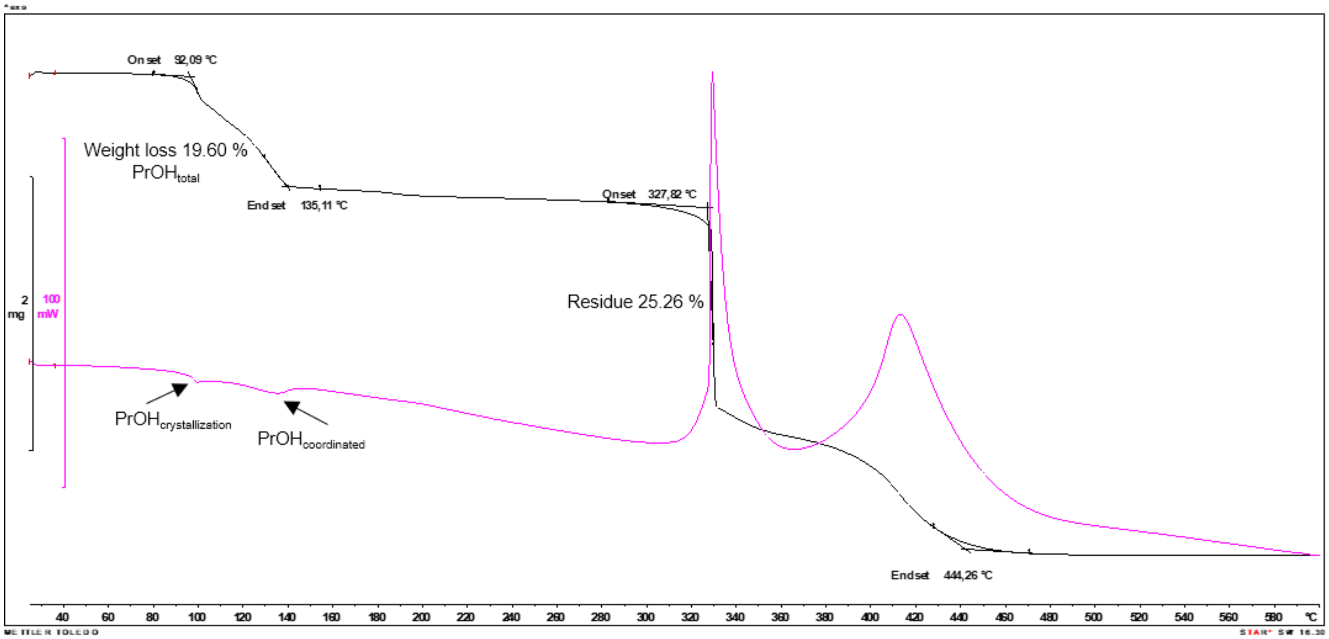

Figure S5. TG-DSC curve for the mononuclear compound $[MoO_2(L^1)(2\text{-}PrOH)]\cdot 2\text{-}PrOH$. Black curve presents weight loss, and pink curve presents heatflow.

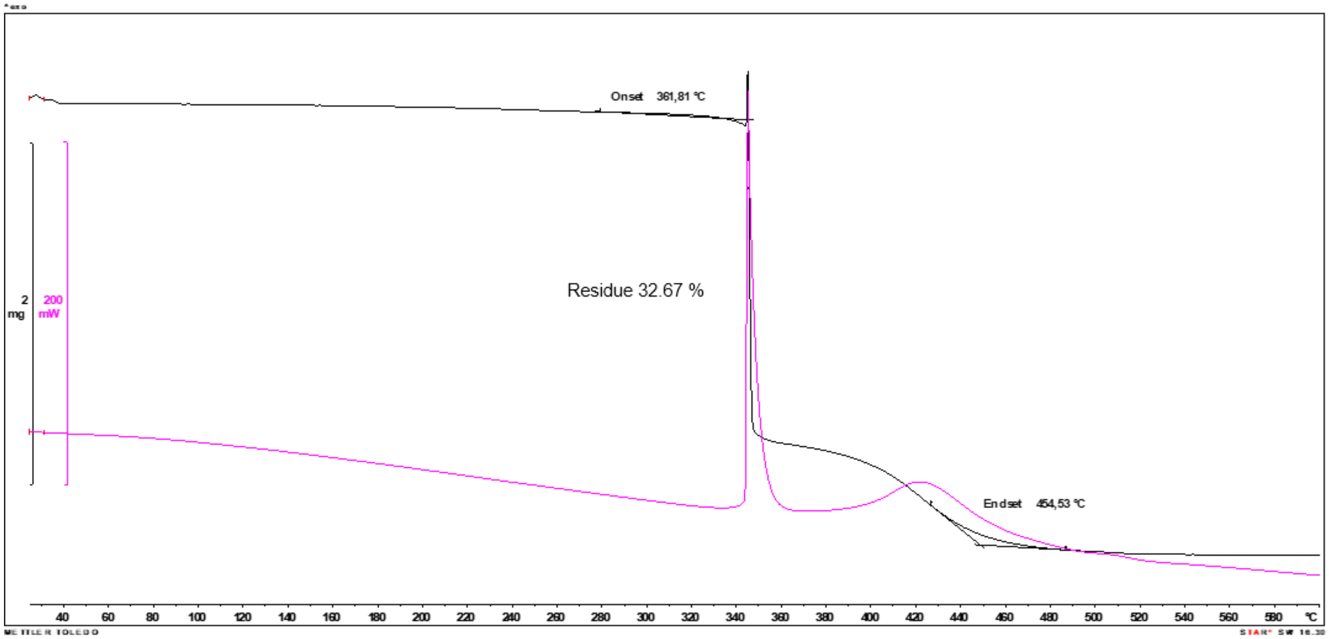

Figure S6. TG-DSC curve for the polynuclear compound $[MoO_2(L^1)]_n$. Black curve presents weight loss, and pink curve presents heatflow.

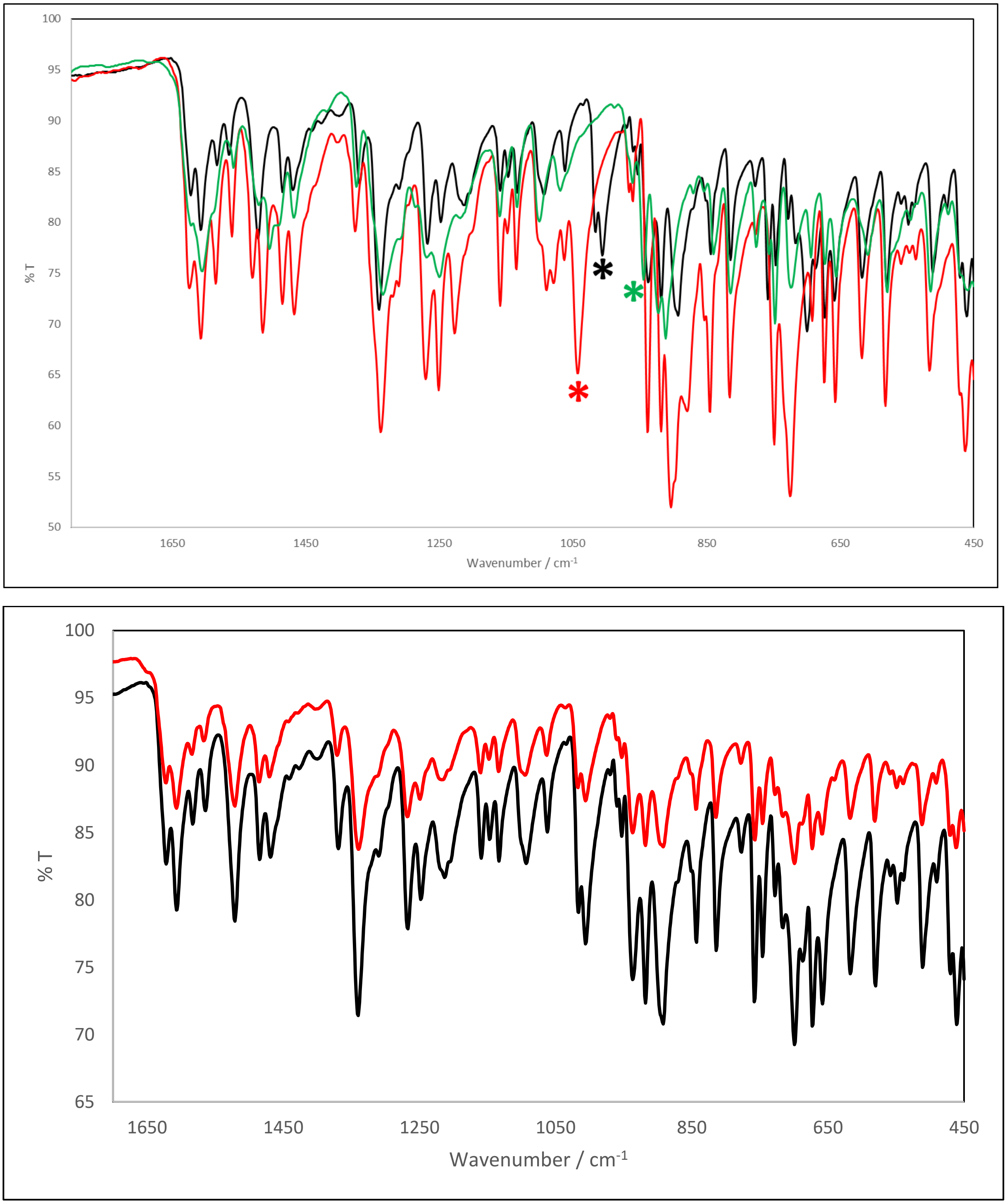

Figure S7 (up and down). Up: Original IR spectra comparison for [$MoO_2(L^1)(MeOH)$] (black), [$MoO_2(L^1)(EtOH)$] (red) and [$MoO_2(L^1)(2\text{-}PrOH)$]·2-PrOH (green). **Down**: According to Table 1, the main text of the manuscript, when the [$MoO_2(L^1)(2\text{-}PrOH)$]·2-PrOH is exposed to the MeOH vapors, the IR of the obtained species (red curve) corresponds to the original IR spectra of the sample[$MoO_2(L^1)(MeOH)$] (black curve). The same behavior is valid for all the tested samples.

Table S1. General and crystal data, summary of intensity data collection and structure refinement for H_2L^2, $[MoO_2(L^1)(H_2O)]$, $[MoO_2(L^2)(H_2O)]$, $[MoO2(L^1)]_n$, $[MoO_2(L^1)(MeOH)]·MeOH$, $[MoO_2(L^1)(EtOH)]·EtOH$, $[MoO_2(L^1)(2\text{-}PrOH)]·2\text{-}PrOH$

**Compound**	**H_2L^2**	**$[MoO_2(L^1)(H_2O)]$**	**$[MoO_2(L^2)(H_2O)]$**	**$[MoO2(L^1)]_n$**	**$[MoO_2(L^1)(MeOH)]·MeOH$**	**$[MoO_2(L^1)(EtOH)]·EtOH,$**	**$[MoO_2(L^1)(2\text{-}PrOH)]·2\text{-}PrOH$**
Chemical formula	$C_{14}H_{11}N_3\ O_5$	$C_{14}H_{11}MoN_3O_8$	$C_{14}H_{11}MoN_3O_8$	$C_{14}H_9MoN_3O_7$	$C_{16}H_{17}MoN_3O_9$	$C_{18}H_{21}MoN_3O_9$	$C_{40}H_{50}Mo_2N_6O_{18}$
M_r	301.26	445.20	445.20	427.18	491.26	519.32	1094.74
Crystal system, color and habit	Monoclinic , yellow, prism	Triclinic, orange, prism	Monoclinic , yellow, prism	Orthorhombic, light brown, prism	Monoclinic, yellow, prism	Monoclinic, orange, plate	Triclinic, yellow, plate
Crystal dimensions / mm^3	0.09x0.14x 0.17	0.02 x 0.03 x 0.12	0.02 x 0.03 x 0.08	0.03 x 0.04 x 0.11	0.03 x 0.06 x 0.58	0.02 x 0.21 x 0.32	0.02 x 0.09 x 0.26
Space group	$P2_1/c$	P-1	$P2_1/n$	$Pbca$	$P2_1/n$	$P2_1/c$	P-1
T/K	170	170	170	170	170	170	170
Z	4	2	4	8	4	4	2
Θ range/°	4.6, 69.0	4.3, 77.6	4.0, 77.8	3.0, 66.0	4.7, 80.1	4.1, 80.0	2.9, 83.4
Index ranges	$-8 \leq h \leq 8$ $-16 \leq k \leq 16$ $-14 \leq l \leq 16$	$-9 \leq h \leq 8$ $-13 \leq k \leq 13$ $-12 \leq l \leq 13$	$-8 \leq h \leq 6$ $-13 \leq k \leq 13$ $-26 \leq l \leq 24$	$-4 \leq h \leq 8$ $-15 \leq k \leq 15$ $-35 \leq l \leq 33$	$-11 \leq h \leq 13$ $-18 \leq k \leq 17$ $-15 \leq l \leq 15$	$-13 \leq h \leq 14$ $-23 \leq k \leq 24$ $-13 \leq l \leq 12$	$-13 \leq h \leq 14$ $-17 \leq k \leq 17$ $-20 \leq l \leq 20$
a /Å	6.8216(4)	7.15637(18)	6.6996(2)	7.6607(3)	10.43886(8)	11.1626(1)	11.6829(2)
b /Å	13.6297(8)	10.3934(3)	10.9278(4)	13.2610(5)	14.23089(14)	19.0265(2)	13.9954(2)
c /Å	13.6932(8)	10.90525(18)	20.7351(8)	29.5416(10)	12.47281(11)	10.1937(1)	15.9802(3)
α/°	90	97.1372(18)	90	90	90	90	80.580(1)
β/°	91.297(6)	107.7335(19)	90.057(3)	90	93.0734(8)	104.797(1)	70.727(2)
γ/°	90	95.086(2)	90	90	90	90	69.613(1)

V /Å^3	1272.82(13)	759.70(3)	1518.06(9)	3001.09(19)	1850.22(3)	2093.19(4)	2308.44(7)
Radiation (Å)	CuKa 1.54184						
D_{calc} /g cm^{-3}	1.572	1.946	1.948	1.891	1.764	1.648	1.575
μ /mm^{-1}	1.039	7.586	7.593	7.600	6.335	5.635	5.141
F(000)	624	444	888	1696	992	1056	1120
No. refined parameters, N_p /restraints	209/3	244/3	238/0	229/1	273/3	288/8	597/10
Reflections collected, unique (R_{int}), observed [$I \geq 2\sigma(I)$]	29825, 4636 (0.105), 4064	11674, 3185 (0.030), 3031	13647, 3090 (0.061), 2444	9518, 2559, (0.064), 1635	27578, 3991, (0.060), 3771	33273, 4523, (0.060), 4271	86133, 9941, (0.055), 9107
R_1[a] [$I \geq 2\sigma(I)$]	0.0585	0.0235	0.0637	0.0863	0.0310	0.0336	0.0870
g_1, g_2 in w[b]	0.1480, 0.1490	0.0338, 0.4842	0.1421,0.3690	0.0587, 64.1565	0.0488, 1.2216	0.0486, 2.0274	0.0113, 54.4194
wR_2[c] (all data)	0.1953	0.0628	0.1880	0.2248	0.0867	0.0912	0.2217
Goodness of fit on F^2, S[d]	1.05	1.08	1.03	1.09	1.10	1.07	1.09
Largest diff. peak/hole / e Å^{-3}	-0.31, 0.23	-0.63, 0.34	-1.51, 3.08	-2.93, 2.75	-0.95, 0.46	-0.83, 0.73	-1.51, 2.97

[a] $R = \Sigma||F_o| - |F_c||/ \Sigma|F_o|$; [b] $w = 1/[\sigma^2(F_o^2) + (g_1P)^2 + g_2P]$ where $P = (F_o^2 + 2F_c^2)/3$

[c] $wR = \{\Sigma[w(F_o^2 - F_c^2)^2]/\Sigma[w(F_o^2)^2]\}^{1/2}$

[d] $S = \{\Sigma[w(F_o^2 - F_c^2)^2]/(N_r - N_p)\}^{1/2}$ where N_r = number of independent reflections, N_p = number of refined parameters.

Description of molecular and crystal structure of H_2L^2

Details of the structure solution: Structure for H_2L^2 was solved as two-component twin. Detailed inspection of its reciprocal space unveiled the case of non-merohedral twinning, with the lattices of the two twin components being visible. The second twin domain was found to be rotated by -180 ° around [-0.00 0.00 1.00] (reciprocal) or [0.05 0.00 1.00] (direct). Decomposition of the twinned data set revealed that the two twin domains are present in the ratio 0.436:0.564. From total of 29825 reflections collected, 12250 were isolated for the first domain, 12291 for the second one, leaving 5284 overlapped reflections. The structure was solved with detwinned hklf4 file of the major twin component and refined with the twinned hklf5 file containing merged data.

H_2L^2 crystalizes in the enol-amino tautomeric form when considering its aldehyde residue, whereas the hydrazone part of the molecule assumes the keto tautomeric form, which is evident from the relevant bond distances (Figure S1; Table S2). Such arrangement is stabilized by intramolecular hydrogen bond of the O–H···N type. The phenyl ring of the aldehyde residue in H_2L^2 is nearly co-planar with the central backbone (C7-N2-N3-C8-O4), while the plane defined by the phenyl ring of the hydrozone residue is inclined from it. In the crystal structure H_2L^2 molecules associate *via* O–H···O and N–H···O hydrogen bonds as well as C–H···O interactions (Figure S3, Table S2).

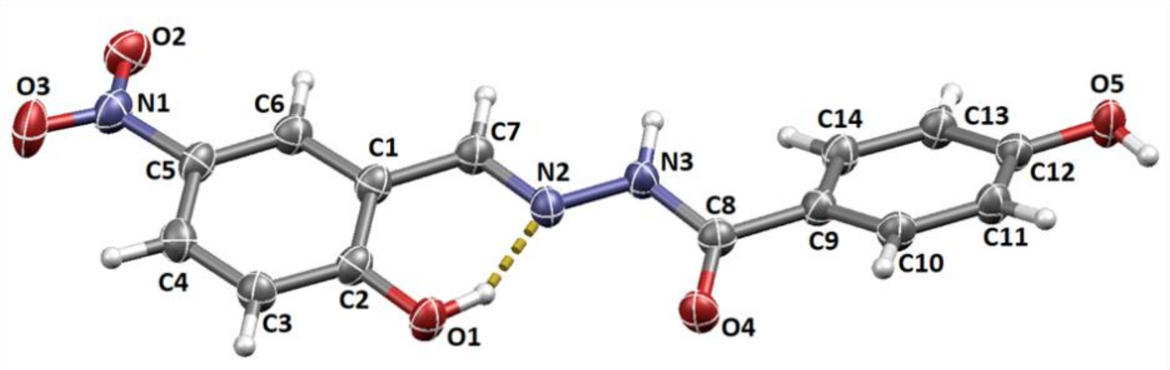

Figure S8. Molecular structures with the atom labeling scheme for H_2L^2. The angle between the LS planes defined by C1-C2-C3-C4-C5-C6 ring and O1-N2-O4 fragment is 5.28(7) °. The angle between the LS planes defined by C1-C2-C3-C4-C5-C6 and C9-C10-C11-C12-C13-C14 rings is 22.83(7) °.

Table S2. Selected geometrical parameters (bond lengths and angles) for H_2L^2

**A–B–C**	***d*(A–B)/Å**	***d*(B–C)/Å**	**∠(A–B–C)/°**
**H_2L^2**			
N3–N2–C7	1.363(3)	1.286(3)	119.23(19)
N2–N3–C8	1.363(3)	1.358(3)	117.58(19)

C2–C1–C7	1.418(3)	1.452(3)	121.9(2)
O1–C2–C1	1.344(3)	1.418(3)	121.9(2)
N3–C8–C9	1.358(3)	1.473(3)	116.44(19)
N2–C7–C1	1.286(3)	1.452(3)	118.6(2)
O4–C8–N3	1.233(3)	1.358(3)	120.6(2)
O4–C8–C9	1.233(3)	1.473(3)	122.9(2)

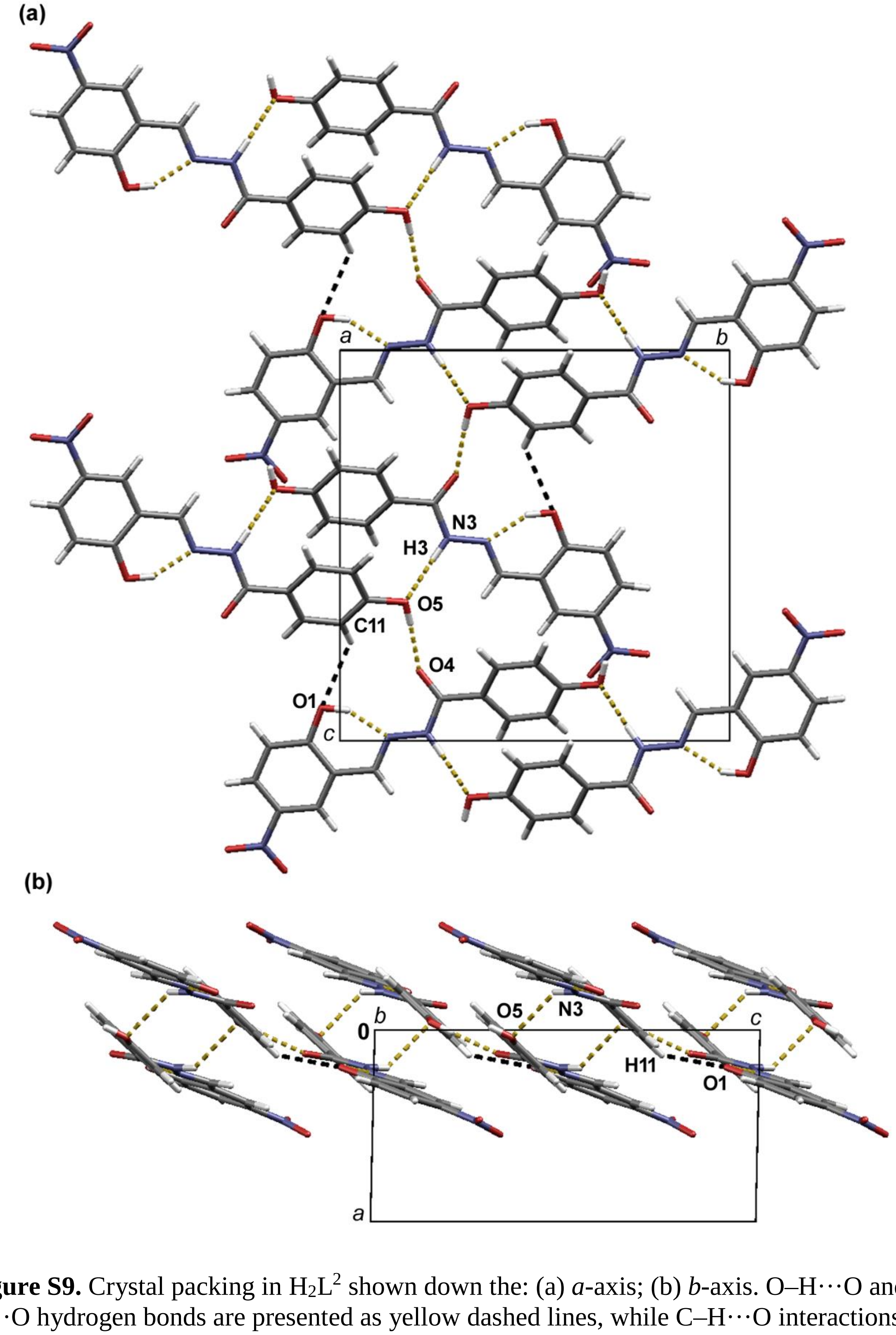

Figure S9. Crystal packing in H_2L^2 shown down the: (a) *a*-axis; (b) *b*-axis. O–H···O and N–H···O hydrogen bonds are presented as yellow dashed lines, while C–H···O interactions are shown as black dashed lines.

Table S3. Geometry of hydrogen bonds and C (Å, º) for H_2L^2

D–H···A	D–H	H···A	D···A	∠D–H···A	Symmetry code
**H_2L^2**					
O1–H1···N2	0.90(2)	1.74(3)	2.569(3)	151(4)	–
N3–H3···O5	0.89(3)	2.45(3)	3.165(3)	138(2)	-*x*,1-*y*,1-*z*
O5–H5···O4	0.82(3)	1.86(3)	2.680(2)	177(5)	-*x*,-1/2+*y*,1/2-*z*
C3–H3A···O5	0.9500	2.5200	3.405(3)	155.00	*x*,1+*y*,*z*
C7–H7··· O3	0.9500	2.3400	3.149(3)	142.00	1-*x*,-1/2+*y*,3/2-*z*
C11–H11··· O1	0.9500	2.5700	3.214(3)	126.00	-*x*,-1/2+*y*,1/2-*z*

Description of molecular and crystal structures of Mo(VI) complexes

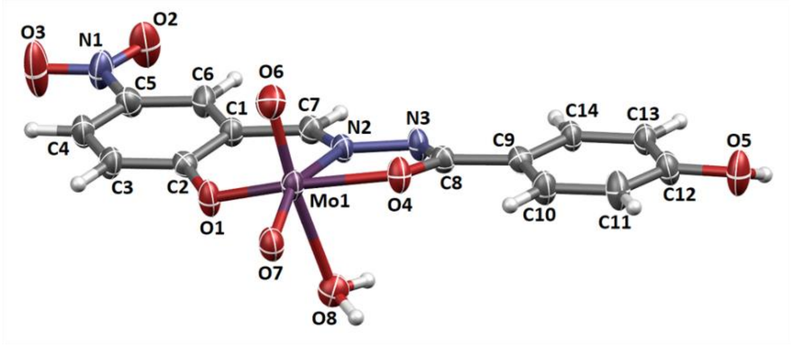

(a)

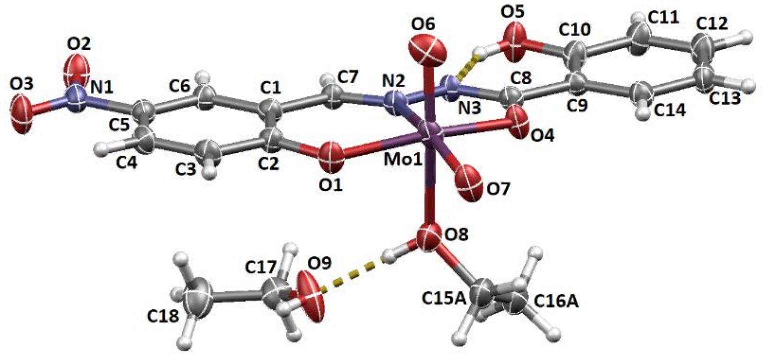

(b)

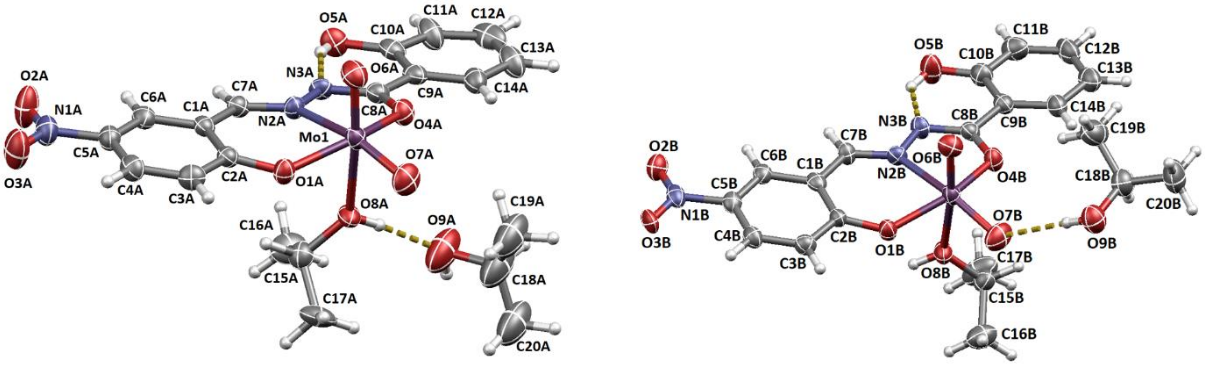

(c)

Figure S10. Molecular structures with the atom labeling scheme for: (a) [MoO_2(L[2])(H_2O)]; (b) [MoO_2(L[1])(EtOH)]·EtOH; and (c) [MoO_2(L[1])(2-PrOH)]·2-PrOH. The displacement ellipsoids are drawn at 50% probability level at 170 K. Hydrogen atoms are presented as spheres of arbitrary small radii. Intramolecular hydrogen bond of the O–H···N type are highlighted by orange dashed line. In (b) only major component of the disordered coordinated ethanol molecule is shown for clarity (the ratio of the occupancies for the two components is ≈ 0.63:0.37). In contrast to remaining structures of Mo(VI) complexes discussed here, the asymmetric unit of [MoO_2(L[1])(2-PrOH)]·2-PrOH contains two coordination moieties and two propanol molecules. For clarity, the molecules in (c) are not shown at their true position within the asymmetric unit.

Table S4. Selected geometrical parameters (bond lengths and angles) for [MoO_2(L^1)(H_2O)], [MoO_2(L^2)(H_2O)], [MoO2(L^1)]$_n$, [MoO_2(L^1)(MeOH)]·MeOH, [MoO_2(L^1)(EtOH)]·EtOH, [MoO_2(L^1)(2-PrOH)]·2-PrOH

**A−B–C**	***d*(A−B)/Å**	***d*(B–C)/Å**	**∠(A–B–C)/°**
**[MoO_2(L^1)(H_2O)]**			
O1–Mo1–O4	1.9505(15)	2.0191(17)	147.89(7)
O1–Mo1–O6	1.9505(15)	1.695(2)	99.24(7)
O1–Mo1–O7	1.9505(15)	1.6976(16)	104.74(7)
O1–Mo1–O8	1.9505(15)	2.290(2)	79.91(7)
O1–Mo1–N2	1.9505(15)	2.2594(18)	80.12(6)
O4–Mo1–O6	2.0191(17)	1.695(2)	97.66(9)
O4–Mo1–O7	2.0191(17)	1.6976(16)	97.19(7)
O4–Mo1–O8	2.0191(17)	2.290(2)	80.04(8)
O4–Mo1–N2	2.0191(17)	2.2594(18)	72.00(6)
O6–Mo1–O7	1.695(2)	1.6976(16)	104.34(10)
O6–Mo1–O8	1.695(2)	2.290(2)	173.05(8)
O6–Mo1–N2	1.695(2)	2.2594(18)	92.64(8)
O7–Mo1–O8	1.6976(16)	2.290(2)	82.50(9)
O7–Mo1–N2	1.6976(16)	2.2594(18)	161.12(9)
O8–Mo1–N2	2.290(2)	2.2594(18)	80.42(7)
Mo1–O1–C2	1.9505(15)	1.336(3)	136.27(14)
Mo1–O4–C8	2.0191(17)	1.315(3)	119.66(13)
Mo1–N2–N3	2.2594(18)	1.391(3)	114.25(13)
Mo1–N2–C7	2.2594(18)	1.285(3)	129.78(15)
N3–N2–C7	1.391(3)	1.285(3)	115.77(18)
N2–N3–C8	1.391(3)	1.305(3)	110.60(18)
O1–C2–C1	1.336(3)	1.415(3)	122.61(19)
N2–C7–C1	1.285(3)	1.450(3)	122.60(19)
C2–C1–C7	1.415(3)	1.450(3)	123.18(19)
N3–C8–C9	1.305(3)	1.470(3)	117.85(19)

O4–C8–N3	1.315(3)	1.305(3)	123.2(2)
O4–C8–C9	1.315(3)	1.470(3)	118.96(18)
**[$MoO_2(L^2)(H_2O)$]**			
O1–Mo1–O4	1.955(6)	1.993(4)	150.5(2)
O1–Mo1–O6	1.955(6)	1.689(5)	98.1(3)
O1–Mo1–O7	1.955(6)	1.715(6)	100.6(2)
O1–Mo1–O8	1.955(6)	2.310(5)	83.9(2)
O1–Mo1–N2	1.955(6)	2.249(6)	80.8(2)
O4–Mo1–O6	1.993(4)	1.689(5)	96.0(2)
O4–Mo1–O7	1.993(4)	1.715(6)	100.6(2)
O4–Mo1–O8	1.993(4)	2.310(5)	79.29(19)
O4–Mo1–N2	1.993(4)	2.249(6)	71.65(18)
O6–Mo1–O7	1.689(5)	1.715(6)	104.8(3)
O6–Mo1–O8	1.689(5)	2.310(5)	172.8(2)
O6–Mo1–N2	1.689(5)	2.249(6)	98.0(2)
O7–Mo1–O8	1.715(6)	2.310(5)	81.5(2)
O7–Mo1–N2	1.715(6)	2.249(6)	156.7(2)
O8–Mo1–N2	2.310(5)	2.249(6)	75.5(2)
Mo1–O1–C2	1.955(6)	1.326(9)	134.2(4)
Mo1–O4–C8	1.993(4)	1.322(7)	121.4(4)
Mo1–N2–N3	2.249(6)	1.404(6)	114.9(4)
Mo1–N2–C7	2.249(6)	1.272(9)	128.5(4)
N3–N2–C7	1.404(6)	1.272(9)	116.6(5)
N2–N3–C8	1.404(6)	1.309(9)	109.7(5)
O1–C2–C1	1.326(9)	1.428(9)	123.1(6)
N2–C7–C1	1.272(9)	1.476(9)	124.7(6)
C2–C1–C7	1.428(9)	1.476(9)	120.8(6)
N3–C8–C9	1.309(9)	1.461(9)	121.7(5)
O4–C8–N3	1.322(7)	1.309(9)	122.0(5)

O4–C8–C9	1.322(7)	1.461(9)	116.3(5)
**[MoO2(L^1)]$_n$**			
O1–Mo1–O4	1.913(6)	1.971(6)	150.8(4)
O1–Mo1–O6	1.913(6)	1.683(9)	99.6(4)
O1–Mo1–O7	1.913(6)	1.680(12)	102.7(4)
O1–Mo1–N2	1.913(6)	2.218(11)	81.5(3)
O1–Mo1–O6^i	1.913(6)	2.163(9)	80.5(3)
O4–Mo1–O6	1.971(6)	1.683(9)	98.3(4)
O4–Mo1–O7	1.971(6)	1.680(12)	97.3(4)
O4–Mo1–N2	1.971(6)	2.218(11)	72.6(3)
O4–Mo1– O6^i	1.971(6)	2.163(9)	81.7(4)
O6–Mo1–O7	1.683(9)	1.680(12)	98.6(7)
O6–Mo1–N2	1.683(9)	2.218(11)	100.6(6)
O6–Mo1– O6^i	1.683(9)	2.163(9)	179.7(7)
O7–Mo1–O6^i	1.680(12)	2.163(9)	81.1(6)
O7–Mo1–N2	1.680(12)	2.218(11)	159.4(6)
O6^i–Mo1–N2	2.163(9)	2.218(11)	79.7(6)
Mo1–O1–C2	1.913(6)	1.355(13)	134.1(7)
Mo1–O4–C8	1.971(6)	1.321(17)	120.9(7)
Mo1–N2–N3	2.218(11)	1.386(14)	113.1(8)
Mo1–N2–C7	2.218(11)	1.268(17)	127.9(9)
N3–N2–C7	1.386(14)	1.268(17)	119.1(12)
N2–N3–C8	1.386(14)	1.274(17)	112.8(12)
O1–C2–C1	1.355(13)	1.373(19)	122.0(9)
N2–C7–C1	1.268(17)	1.439(17)	124.5(14)
C2–C1–C7	1.373(19)	1.439(17)	123.0(12)
N3–C8–C9	1.274(17)	1.469(17)	119.1(12)
O4–C8–N3	1.321(17)	1.274(17)	120.6(11)
O4–C8–C9	1.321(17)	1.469(17)	117.2(11)

[i] 1/2+x,y,3/2-z; ∠(Mo1–O6–Mo1[i]) = 169.6(7)°			
**$MoO_2(L^1)(MeOH)]\cdot MeOH$**			
O1–Mo1–O4	1.9391(16)	2.0028(17)	148.12(7)
O1–Mo1–O6	1.9391(16)	1.6941(19)	99.19(8)
O1–Mo1–O7	1.9391(16)	1.7135(19)	105.36(8)
O1–Mo1–O8	1.9391(16)	2.2784(19)	79.20(7)
O1–Mo1–N2	1.9391(16)	2.252(2)	80.74(7)
O4–Mo1–O6	2.0028(17)	1.6941(19)	98.27(8)
O4–Mo1–O7	2.0028(17)	1.7135(19)	95.69(8)
O4–Mo1–O8	2.0028(17)	2.2784(19)	79.08(7)
O4–Mo1–N2	2.0028(17)	2.252(2)	72.15(7)
O6–Mo1–O7	1.6941(19	1.7135(19)	104.67(9)
O6–Mo1–O8	1.6941(19	2.2784(19)	170.22(8)
O6–Mo1–N2	1.6941(19	2.252(2)	92.13(8)
O7–Mo1–O8	1.7135(19)	2.2784(19)	85.02(8)
O7–Mo1–N2	1.7135(19)	2.252(2)	160.70(8)
O8–Mo1–N2	2.2784(19)	2.252(2)	78.09(7)
Mo1–O1–C2	1.9391(16)	1.342(3)	134.29(14)
Mo1–O4–C8	2.0028(17)	1.323(3)	119.89(15)
Mo1–N2–N3	2.252(2)	1.389(3)	114.43(14)
Mo1–N2–C7	2.252(2)	1.279(3)	128.67(15)
N3–N2–C7	1.389(3)	1.279(3)	116.87(19)
N2–N3–C8	1.389(3)	1.300(3)	110.53(18)
O1–C2–C1	1.342(3)	1.410(3)	122.48(19)
N2–C7–C1	1.279(3)	1.453(3)	123.3(2)
C2–C1–C7	1.410(3)	1.453(3)	122.7(2)
N3–C8–C9	1.300(3)	1.465(3)	119.5(2)
O4–C8–N3	1.323(3)	1.300(3)	122.9(2)
O4–C8–C9	1.323(3)	1.465(3)	117.6(2)

MoO$_2$(L^1)(EtOH)]·EtOH			
O1–Mo1–O4	1.9361(17)	2.0084(18)	149.88(7)
O1–Mo1–O6	1.9361(17)	1.693(2)	98.78(9)
O1–Mo1–O7	1.9361(17)	1.7082(19)	103.31(8)
O1–Mo1–O8	1.9361(17)	2.293(2)	80.71(8)
O1–Mo1–N2	1.9361(17)	2.2402(18)	81.17(7)
O4–Mo1–O6	2.0084(18)	1.693(2)	96.63(9)
O4–Mo1–O7	2.0084(18)	1.7082(19)	97.19(8)
O4–Mo1–O8	2.0084(18)	2.293(2)	79.09(8)
O4–Mo1–N2	2.0084(18)	2.2402(18)	72.11(7)
O6–Mo1–O7	1.693(2)	1.7082(19)	105.63(10)
O6–Mo1–O8	1.693(2)	2.293(2)	168.43(9)
O6–Mo1–N2	1.693(2)	2.2402(18)	93.75(8)
O7–Mo1–O8	1.7082(19)	2.293(2)	85.64(9)
O7–Mo1–N2	1.7082(19)	2.2402(18)	158.99(9)
O8–Mo1–N2	2.293(2)	2.2402(18)	74.73(7)
Mo1–O1–C2	1.9361(17)	1.329(3)	137.54(14)
Mo1–O4–C8	2.0084(18)	1.317(3)	119.75(14)
Mo1–N2–N3	2.2402(18)	1.392(2)	114.73(13)
Mo1–N2–C7	2.2402(18)	1.289(3)	129.53(15)
N3–N2–C7	1.392(2)	1.289(3)	115.73(17)
N2–N3–C8	1.392(2)	1.303(3)	110.27(17)
O1–C2–C1	1.329(3)	1.418(3)	122.13(19)
N2–C7–C1	1.289(3)	1.444(3)	123.04(19)
C2–C1–C7	1.418(3)	1.444(3)	123.57(18)
N3–C8–C9	1.303(3)	1.463(3)	119.58(19)
O4–C8–N3	1.317(3)	1.303(3)	122.86(19)
O4–C8–C9	1.317(3)	1.463(3)	117.55(18)
**[MoO$_2$(L^1)(2-PrOH)]·(2-PrOH)**			

O1A–Mo1A–O4A	1.941(7)	1.993(7)	148.6(3)
O1A–Mo1A–O6A	1.941(7)	1.675(7)	99.1(3)
O1A–Mo1A–O7A	1.941(7)	1.675(7)	100.7(4)
O1A–Mo1A–O8A	1.941(7)	2.315(6)	81.6(3)
O1A–Mo1A–N2A	1.941(7)	1.993(7)	80.6(3)
O4A–Mo1A–O6A	1.993(7)	1.675(7)	98.1(4)
O4A–Mo1A–O7A	1.993(7)	1.675(7)	99.9(4)
O4A–Mo1A–O8A	1.993(7)	2.315(6)	76.9(3)
O4A–Mo1A–N2A	1.993(7)	1.993(7)	72.2(3)
O6A–Mo1A–O7A	1.675(7)	1.675(7)	105.1(4)
O6A–Mo1A–O8A	1.675(7)	2.315(6)	169.4(3)
O6A–Mo1A–N2A	1.675(7)	1.993(7)	94.1(3)
O7A–Mo1A–O8A	1.714(9)	2.315(6)	85.1(3)
O7A–Mo1A–N2A	1.714(9)	1.993(7)	160.2(3)
O8A–Mo1A–N2A	2.315(6)	1.993(7)	75.5(3)
Mo1A–O1A–C2A	1.941(7)	1.336(11)	135.3(7)
Mo1A–O4A–C8A	1.993(7)	1.329(12)	120.7(6)
Mo1A–N2A–N3A	1.993(7)	1.389(12)	114.5(5)
Mo1A–N2A–C7A	1.993(7)	1.289(11)	129.1(7)
N3A–N2A–C7A	1.389(12)	1.289(11)	116.3(8)
N2A–N3A–C8A	1.389(12)	1.310(11)	110.8(8)
O1A–C2A–C1A	1.336(11)	1.416(13)	121.7(8)
N2A–C7A–C1A	1.289(11)	1.446(13)	122.8(8)
C2A–C1A–C7A	1.416(13)	1.446(13)	123.4(7)
N3A–C8A–C9A	1.310(11)	1.459(14)	120.4(9)
O4A–C8A–N3A	1.329(12)	1.310(11)	117.8(8)
O4A–C8A–C9A	1.329(12)	1.459(14)	121.7(9)
O1B–Mo1B–O4B	1.952(7)	2.013(7)	149.1(3)
O1B–Mo1B–O6B	1.952(7)	1.678(7)	99.3(3)

O1B–Mo1B–O7B	1.952(7)	1.717(9)	102.7(4)
O1B–Mo1B–O8B	1.952(7)	2.324(7)	80.0(3)
O1B–Mo1B–N2B	1.952(7)	2.230(8)	81.2(3)
O4B–Mo1B–O6B	2.013(7)	1.678(7)	97.5(4)
O4B–Mo1B–O7B	2.013(7)	1.717(9)	97.2(4)
O4B–Mo1B–O8B	2.013(7)	2.324(7)	78.6(3)
O4B–Mo1B–N2B	2.013(7)	2.230(8)	72.1(3)
O6B–Mo1B–O7B	1.678(7)	1.717(9)	106.2(4)
O6B–Mo1B–O8B	1.678(7)	2.324(7)	169.1(3)
O6B–Mo1B–N2B	1.678(7)	2.230(8)	93.4(3)
O7B–Mo1B–O8B	1.717(9)	2.324(7)	84.5(3)
O7B–Mo1B–N2B	1.717(9)	2.230(8)	158.9(3)
O8++--B–Mo1B–N2B	2.324(7)	2.230(8)	75.7(3)
Mo1B–O1B–C2B	1.952(7)	1.326(10)	135.1(7)
Mo1B–O4B–C8B	2.013(7)	1.315(12)	119.4(6)
Mo1B–N2B–N3B	2.230(8)	1.388(12)	115.2(5)
Mo1B–N2B–C7B	2.230(8)	1.298(11)	128.9(7)
N3B–N2B–C7B	1.388(12)	1.298(11)	115.9(8)
N2B–N3B–C8B	1.388(12)	1.297(11)	110.1(8)
O1B–C2B–C1B	1.326(10)	1.401(13)	123.0(8)
N2B–C7B–C1B	1.298(11)	1.451(14)	122.7(9)
C2B–C1B–C7B	1.401(13)	1.451(14)	123.5(8)
N3B–C8B–C9B	1.297(11)	1.476(14)	120.2(9)
O4B–C8B–N3B	1.315(12)	1.297(11)	123.2(9)
O4B–C8B–C9B	1.315(12)	1.476(14)	116.6(8)

Table S5. Selected interplanar angles (in °) for $MoO_2(L^1)(H_2O)$], [$MoO_2(L^2)(H_2O)$], [MoO2(L^1)]$_n$, [$MoO_2(L^1)$(MeOH)]·MeOH, [$MoO_2(L^1)$(EtOH)]·EtOH, [$MoO_2(L^1)$(2-PrOH)]·(2-PrOH). *I* represents LS plane passing through the aldehyde subunit (defined by the C1-C2-C3-C4-C5-C6 atoms), while *II* represents LS plane passing through the aryl ring subunit of the hydrazone part of the molecule (defined by the C9-C10-C11-C12-C13-C14 atoms). L1 represents plane passing through O1, N2 and O4 (chelating atoms). Atoms are numerated according to Figures 1 and 2 and Figure S3.

Interplanar angle	**$MoO_2(L^1)(H_2O)$]**	**[$MoO_2(L^2)(H_2O)$]**	**[MoO2(L^1)]$_n$**	**[$MoO_2(L^1)$(MeOH)]·MeOH**	**[$MoO_2(L^1)$(EtOH)]·EtOH**	**[$MoO_2(L^1)$(2-PrOH)]·2-PrOH**
I-II/	6.81(8)	5.3(3)	5.5(5)	1.71(9)	2.73(9)	6.6(3)-molecule A<br>10.3(3)- molecule B
I–L_1	5.14(8)	13.6(2)	10.3(4)	10.99(7)	2.52(8)	6.1(3)-molecule A<br>4.4(2)-molecule B
L_1–***II***	10.73(10)	12.7(2)	7.8(4)	12.48(8)	1.95(10)	8.2(3)-molecule A<br>9.7(4)- molecule B
$d(L_1$– Mo)/Å	0.3382(17)	0.223(5)	0.279(8)	0.3505(16)	0.2918(19)	0.325(6)-molecule A<br>0.327(6)-molecule B

Table S6. Geometry of hydrogen bonds and C (Å, º) for [MoO_2(L^1)(H_2O)], [MoO_2(L^2)(H_2O)], [MoO2(L^1)]$_n$, [MoO_2(L^1)(MeOH)]·MeOH, [MoO_2(L^1)(EtOH)]·EtOH, [MoO_2(L^1)(2-PrOH)]·2-PrOH

D–H···A	D–H	H···A	D···A	∠D–H···A	Symmetry code
**[MoO_2(L^1)(H_2O)]**					
O5–H5···N3	0.83(2)	1.85(2)	2.579(2)	147(3)	–
O5–H5···O6	0.83(2)	2.34(3)	2.836(3)	120(2)	1-x,1-y,1-z
O8–H8A···O3	0.81(2)	2.10(2)	2.869(3)	160(3)	1+x,y,1+z
O8–H8B···O5	0.83(3)	1.92(3)	2.741(3)	173(4)	2-x,1-y,1-z
C7–H7···O6	0.9500	2.5300	3.153(3)	124.00	1-x,1-y,1-z
C13–H13···O4	0.9500	2.5500	3.331(3)	139.00	2-x,1-y,2-z
**[MoO_2(L^2)(H_2O)]**					
O5–H5···O7	0.8400	1.9300	2.739(7)	162.00	x,1+y,z
O8–H8A···N3	0.8800	2.0400	2.817(8)	146.00	1-x,1-y,1-z
O8–H8B···O2	0.8800	2.1500	2.983(9)	158.00	-1/2+x,1/2-y,-1/2+z
C4–H4···O2	0.9500	2.5800	3.514(9)	167.00	3/2-x,-1/2+y,3/2-z
C6–H6···O3	0.9500	2.3000	3.182(9)	154.00	3/2-x,1/2+y,3/2-z
C7–H7···O3	0.9500	2.5100	3.347(9)	147.00	3/2-x,1/2+y,3/2-z
C10–H10···O5	0.9500	2.3100	3.162(10)	149.00	3/2-x,-1/2+y,1/2-z
C11–H11···O7	0.9500	2.4600	3.362(12)	158.00	3/2-x,-1/2+y,1/2-z
C14–H14···O6	0.9500	2.4000	3.212(10)	143.00	2-x,1-y,1-z
C14–H14···O8	0.9500	2.5500	3.355(10)	142.00	1-x,1-y,1-z
**[MoO2(L^1)]$_n$**					
O5–H5···N3	0.85(17)	1.8(2)	2.647(16)	165.00	-
C7–H7···O7	0.9500	2.5300	2.99(2)	110.00	1-x,-1/2+y,3/2-z
C7–H7···O7	0.9500	2.4500	3.16(2)	131.00	3/2-x,1/2+y,z
**[MoO_2(L^1)(MeOH)]·MeOH**					

O5–H5···N3	0.85(2)	1.93(2)	2.651(3)	143(3)	–
O5–H5···O6	0.85(2)	2.55(4)	3.085(3)	123(3)	1-x,1-y,1-z
O8–H8···O9	0.85(3)	1.78(3)	2.618(3)	173(4)	–
O9–H9···O7	0.81(3)	2.14(3)	2.910(3)	159(4)	3/2-x,-1/2+y,1/2-z
C3–H3···O5	0.9500	2.4900	3.364(3)	153.00	1+x,y,z
**$[MoO_2(L^1)(EtOH)]\cdot EtOH$**					
O5–H5···N3	0.84(3)	1.90(3)	2.619(2)	144(4)	–
O8–H8···O9	0.879(16)	1.715(18)	2.589(3)	172(3)	–
O9–H9···O7	0.83(3)	1.97(3)	2.790(3)	171(5)	x,1/2-y,1/2+z
C3–H3···O6	0.9500	2.5200	3.242(3)	133.00	x,1/2-y,1/2+z
C7–H7···O2	0.9500	2.5100	3.196(3)	130.00	1-x,1-y,2-z
C7–H7···O6	0.9500	2.4700	3.200(3)	134.00	1-x,1-y,1-z
C11–H11···O3	0.9500	2.5800	3.409(3)	145.00	1-x,1/2+y,3/2-z
**$[MoO_2(L^1)(2\text{-}PrOH)]\cdot 2\text{-}PrOH$**					
O5A–H5A···N3A	0.84(11)	1.85(12)	2.638(12)	156(12)	–
O5B–H5B···N3B	0.83(11)	1.98(12)	2.629(12)	134(13)	–
O8A–H8A···O9A	0.86(4)	1.80(3)	2.652(12)	172(11)	–
O8B–H8B···O3B	0.85(6)	1.93(6)	2.776(9)	174(6)	-x,2-y,1-z
O9A–H9A···O9B	0.84(14)	2.02(18)	2.717(15)	140.00	–
O9B–H9B···O7B	0.84(9)	2.07(8)	2.904(11)	175(17)	–
C6B–H6B···O7A	0.9500	2.5900	3.230(12)	125.00	x,1+y,z
C7B–H7B···O7A	0.9500	2.3900	3.128(11)	134.00	x,1+y,z
C7B–H7B···O6A	0.9500	2.5700	3.308(14)	134.00	1-x,1-y,1-z

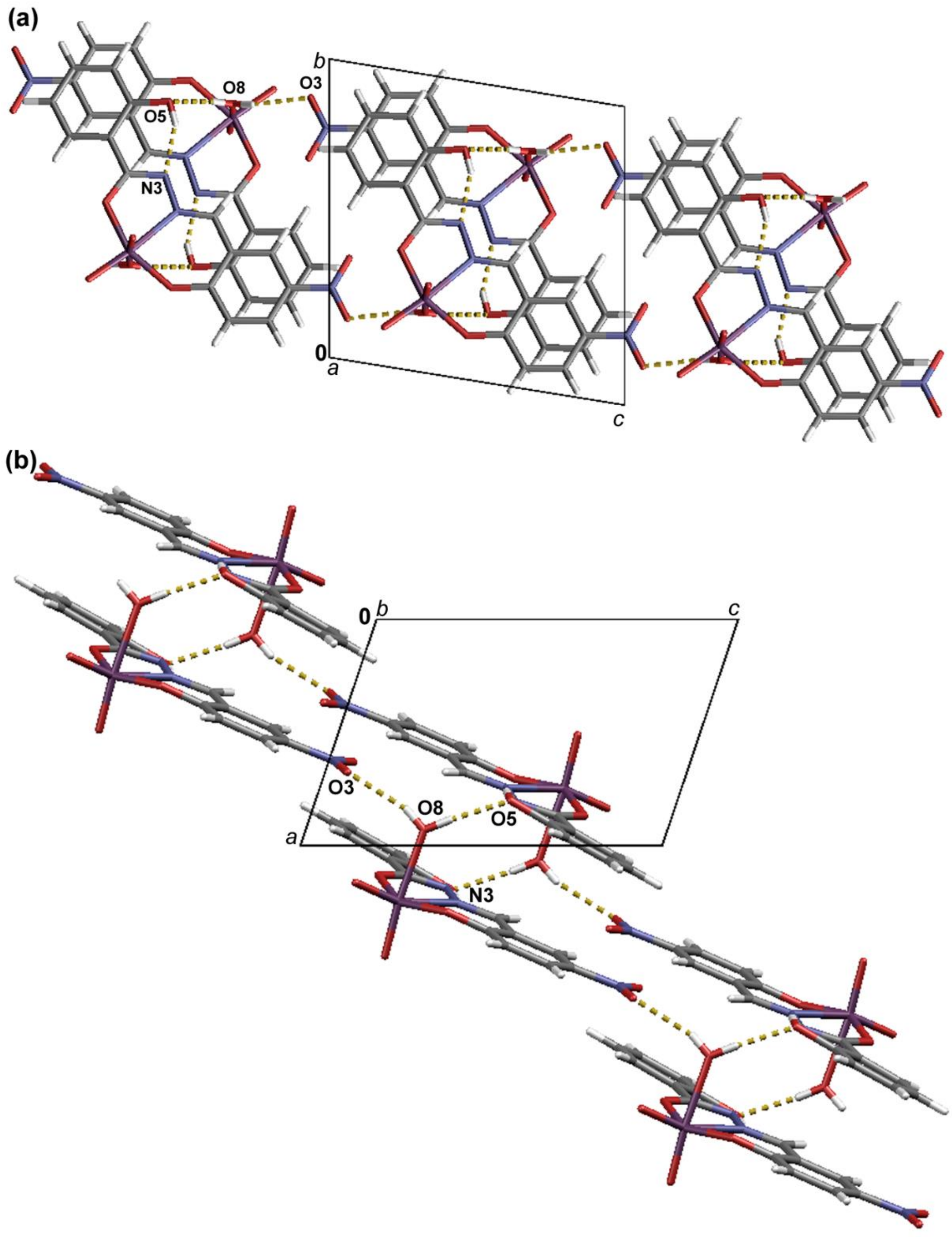

Figure S11. Crystal packing in [$MoO_2(L^1)(H_2O)$] shown down the: (a) *a*-axis; (b) *b*-axis. O–H···O and N–H···O hydrogen bonds are presented as yellow dashed lines.

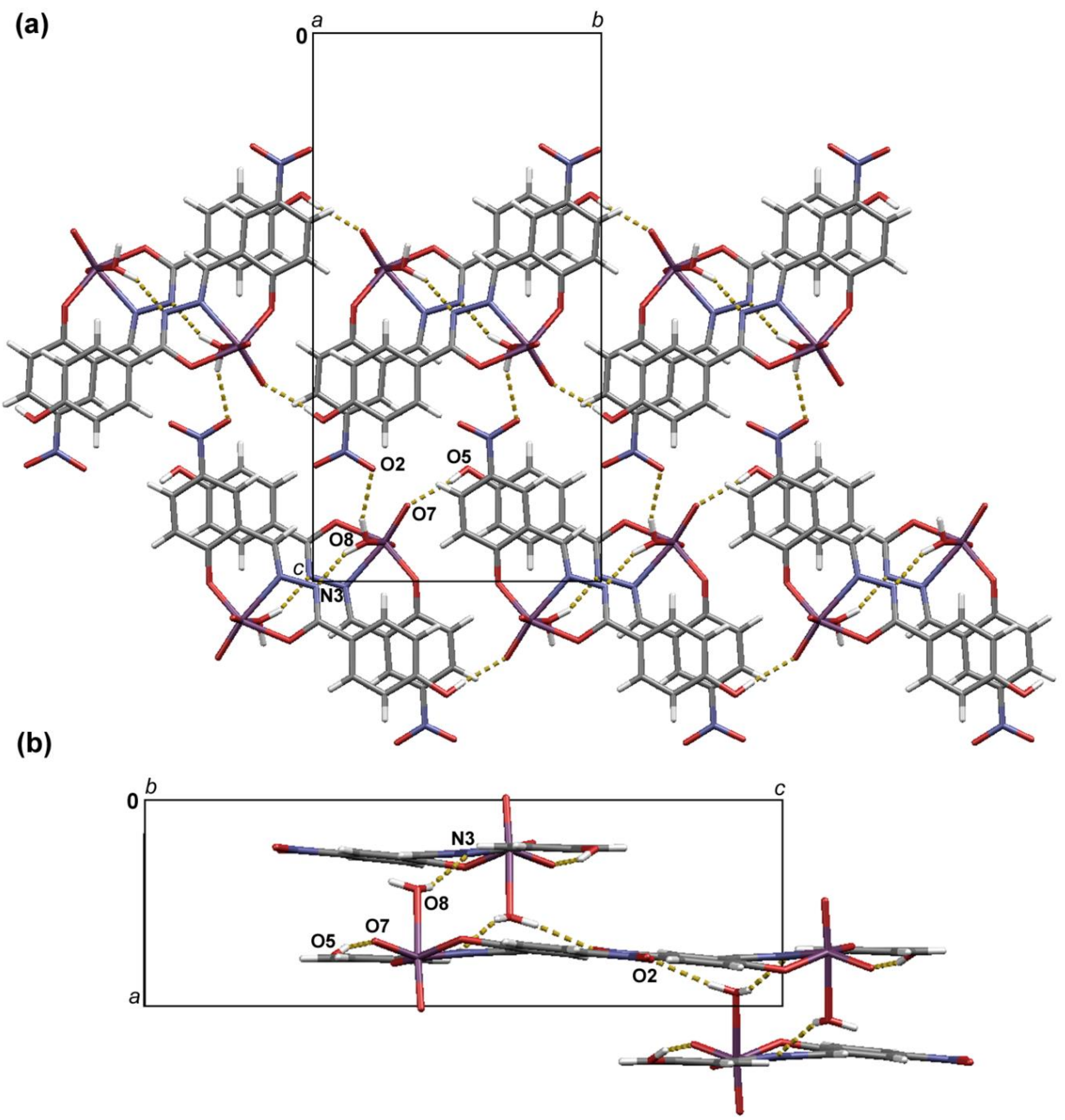

Figure S12. Crystal packing in [MoO_2(L^2)(H_2O)] shown down the: (a) a-axis; (b) b-axis. O–H···O and N–H···O hydrogen bonds are shown as yellow dashed lines.

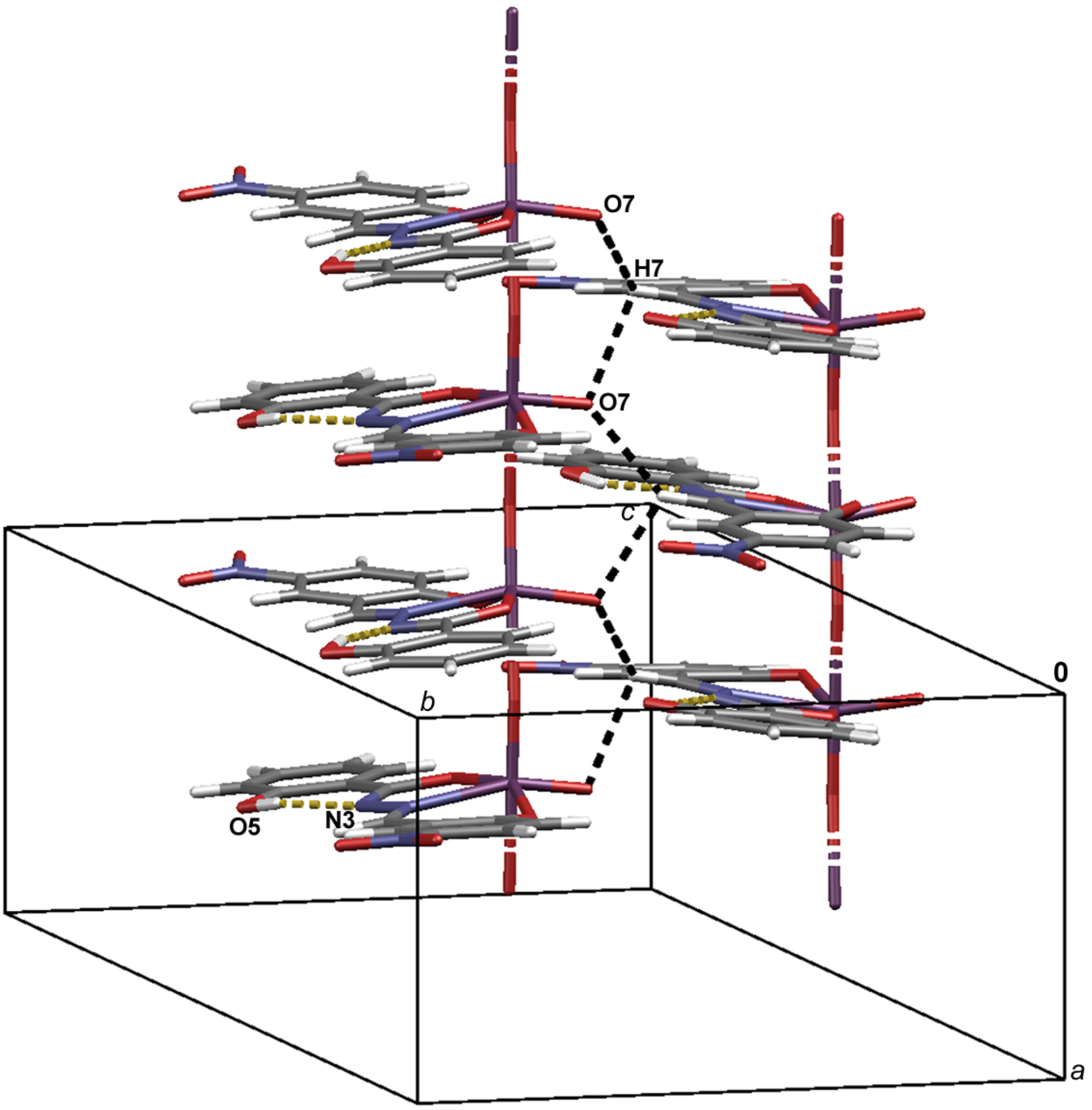

Figure S13. Crystal packing in $[MoO_2(L^1)]_n$. N–H···O hydrogen bonds are presented as yellow dashed lines, while the C–H···O interactions are shown as black dashed lines.

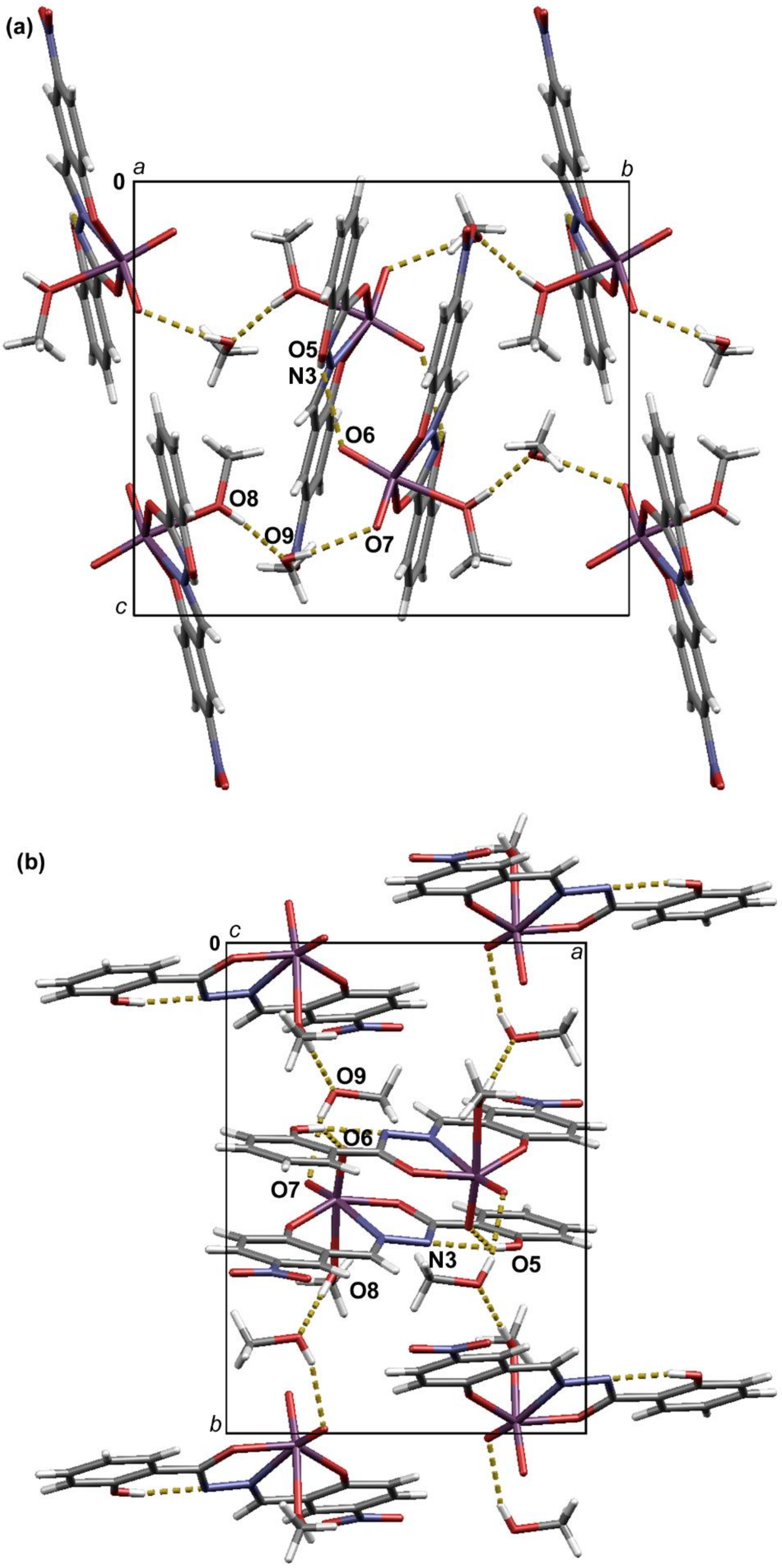

Figure S14. Crystal packing in $MoO_2(L^1)(MeOH)]\cdot MeOH$ shown down the: (a) *a*-axis; (b) *c*-axis. N–H···O and O–H···O hydrogen bonds are presented as yellow dashed lines.

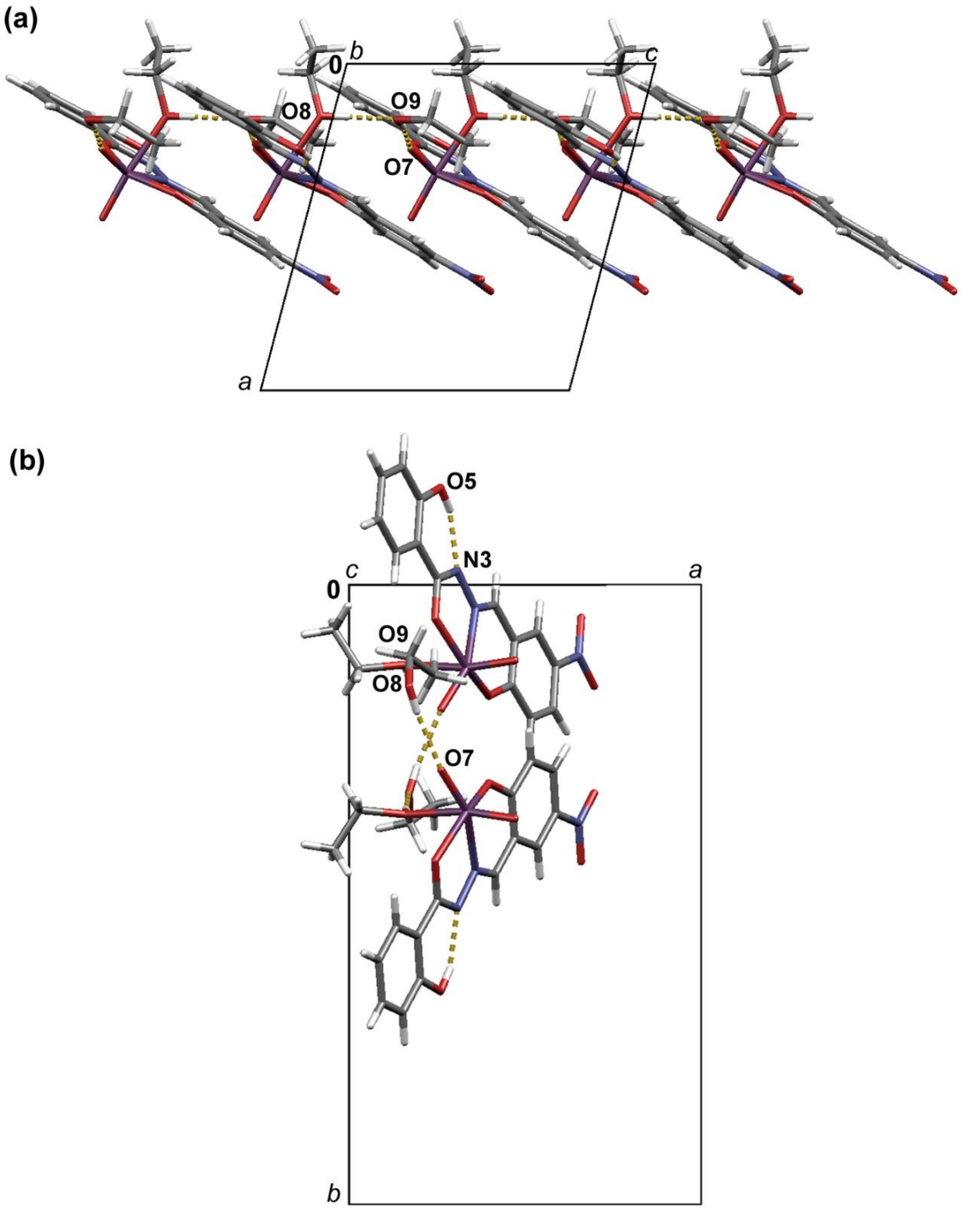

Figure S15. Crystal packing in $MoO_2(L^1)(EtOH)]\cdot EtOH$ shown down the: (a) *b*-axis; (b) *c*-axis. N–H···O and O–H···O hydrogen bonds are presented as yellow dashed lines.

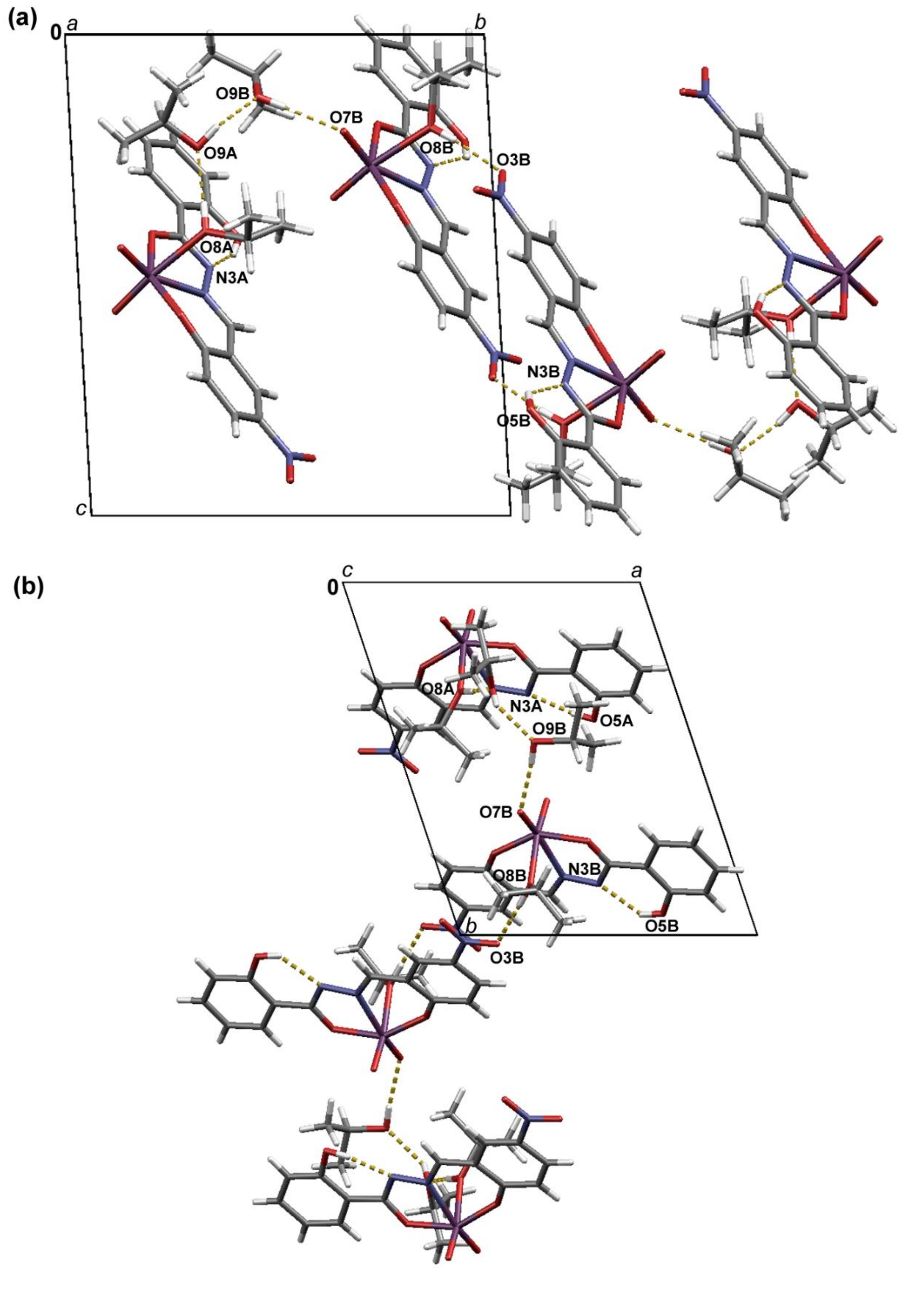

Figure S16. Crystal packing in $[MoO_2(L^1)(2\text{-}PrOH)]\cdot 2\text{-}PrOH$ shown down the: (a) *a*-axis; (b) *c*-axis. N–H···O and O–H···O hydrogen bonds are presented as yellow dashed lines.

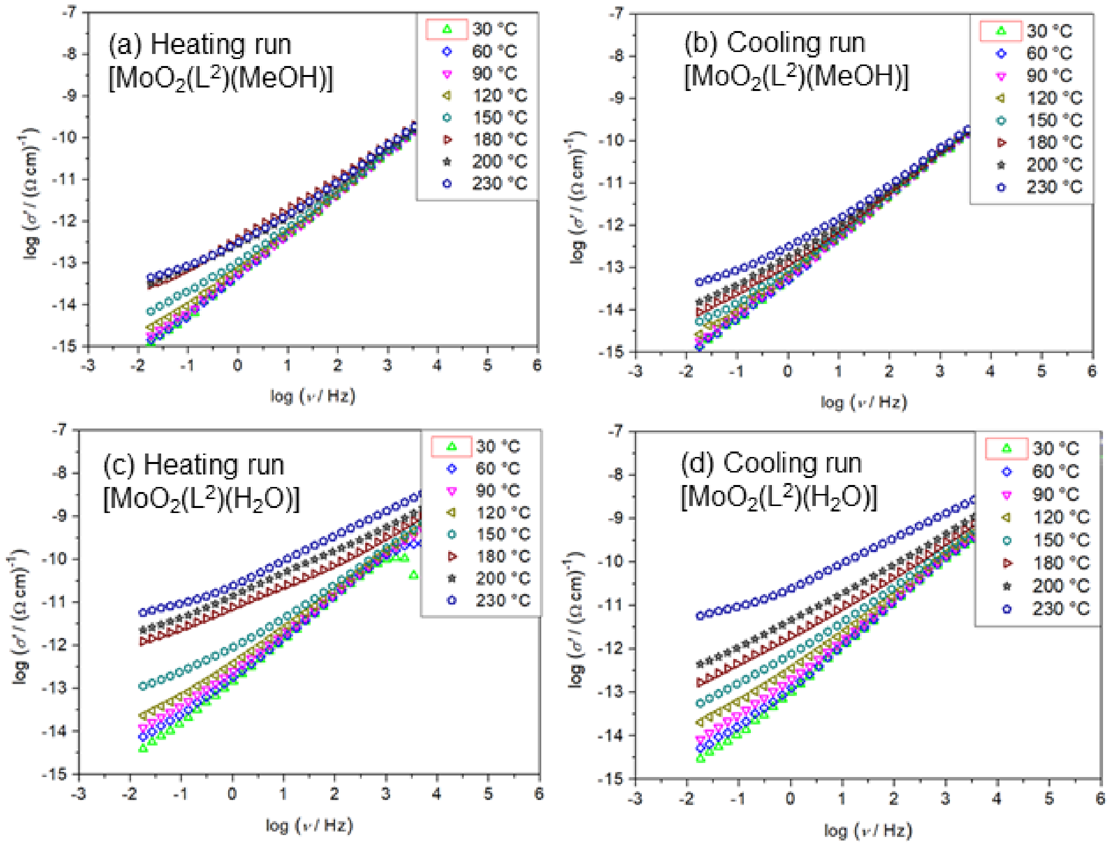

Figure S17. Conductivity spectra for (a-b) $[MoO_2(L^2)(MeOH)]$ and (c-d) $[MoO_2(L^2)(H_2O)]$ in heating (a,c) and cooling (b,d) run.
